# 4-Deoxy-4-fluoro-GalNAz (4FGalNAz) Is a Metabolic Chemical Reporter of O-GlcNAc Modifications, Highlighting the Notable Substrate Flexibility of O-GlcNAc Transferase

**DOI:** 10.1021/acschembio.1c00818

**Published:** 2021-12-21

**Authors:** Emma G. Jackson, Giuliano Cutolo, Bo Yang, Nageswari Yarravarapu, Mary W. N. Burns, Ganka Bineva-Todd, Chloë Roustan, James B. Thoden, Halley M. Lin-Jones, Toin H. van Kuppevelt, Hazel M. Holden, Benjamin Schumann, Jennifer J. Kohler, Christina M. Woo, Matthew R. Pratt

**Affiliations:** † Departments of Chemistry, University of Southern California, Los Angeles, California 90089, United States; ‡ Department of Chemistry and Chemical Biology, Harvard University, Cambridge, Massachusetts 02138, United States; § Department of Biochemistry, University of Texas Southwestern Medical Center, Dallas, Texas 75390, United States; ∥ Chemical Glycobiology Laboratory, The Francis Crick Institute, NW1 1AT London, United Kingdom; ⊥ Structural Biology Science Technology Platform, The Francis Crick Institute, NW1 1AT London, United Kingdom; # Department of Biochemistry, University of Wisconsin, Madison, Wisconsin 53706, United States; ¶ Department of Biochemistry, Radboud Institute for Molecular Life Sciences, Radboud University Medical Centre, 6500 HB Nijmegen The Netherlands; ∇ Department of Chemistry, Imperial College London, W120BZ London, United Kingdom; ○ Biological Sciences, University of Southern California, Los Angeles, California 90089, United States

## Abstract

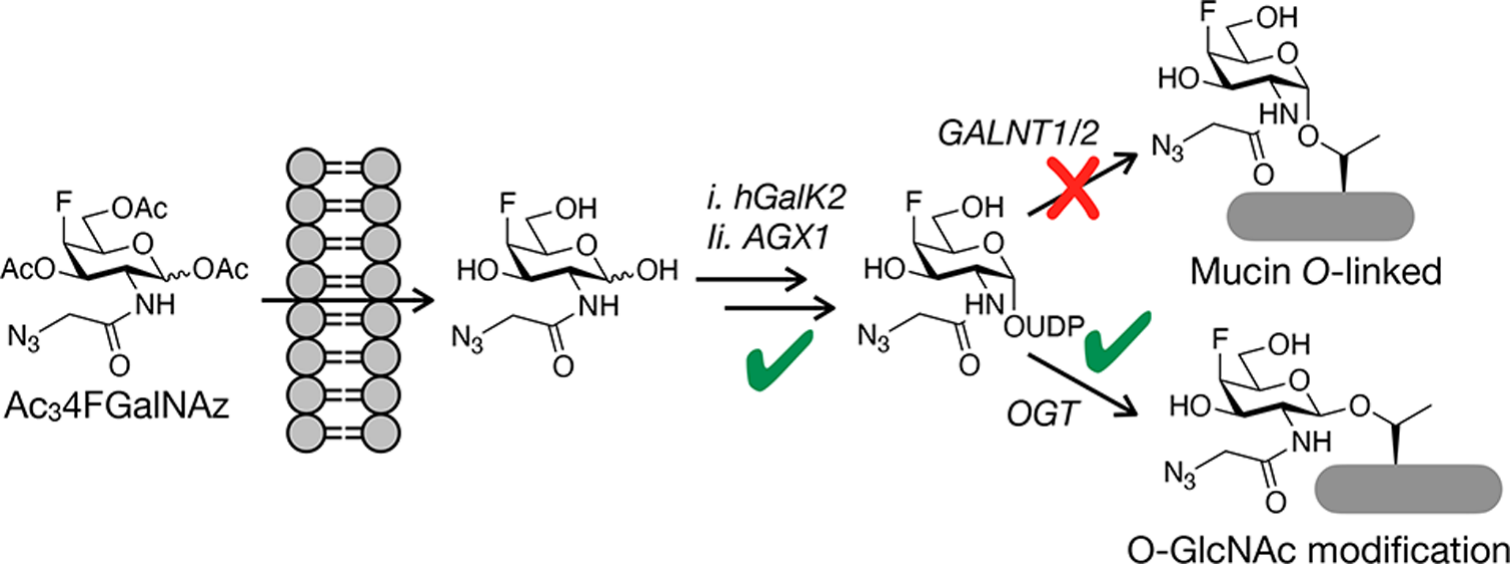

Bio-orthogonal chemistries have revolutionized many fields. For example, metabolic chemical reporters (MCRs) of glycosylation are analogues of monosaccharides that contain a bio-orthogonal functionality, such as azides or alkynes. MCRs are metabolically incorporated into glycoproteins by living systems, and bio-orthogonal reactions can be subsequently employed to install visualization and enrichment tags. Unfortunately, most MCRs are not selective for one class of glycosylation (e.g., N-linked vs O-linked), complicating the types of information that can be gleaned. We and others have successfully created MCRs that are selective for intracellular O-GlcNAc modification by altering the structure of the MCR and thus biasing it to certain metabolic pathways and/or O-GlcNAc transferase (OGT). Here, we attempt to do the same for the core GalNAc residue of mucin O-linked glycosylation. The most widely applied MCR for mucin O-linked glycosylation, GalNAz, can be enzymatically epimerized at the 4-hydroxyl to give GlcNAz. This results in a mixture of cell-surface and O-GlcNAc labeling. We reasoned that replacing the 4-hydroxyl of GalNAz with a fluorine would lock the stereochemistry of this position in place, causing the MCR to be more selective. After synthesis, we found that 4FGalNAz labels a variety of proteins in mammalian cells and does not perturb endogenous glycosylation pathways unlike 4FGalNAc. However, through subsequent proteomic and biochemical characterization, we found that 4FGalNAz does not widely label cell-surface glycoproteins but instead is primarily a substrate for OGT. Although these results are somewhat unexpected, they once again highlight the large substrate flexibility of OGT, with interesting and important implications for intracellular protein modification by a potential range of abiotic and native monosaccharides.

## Introduction

**Figure 1:**
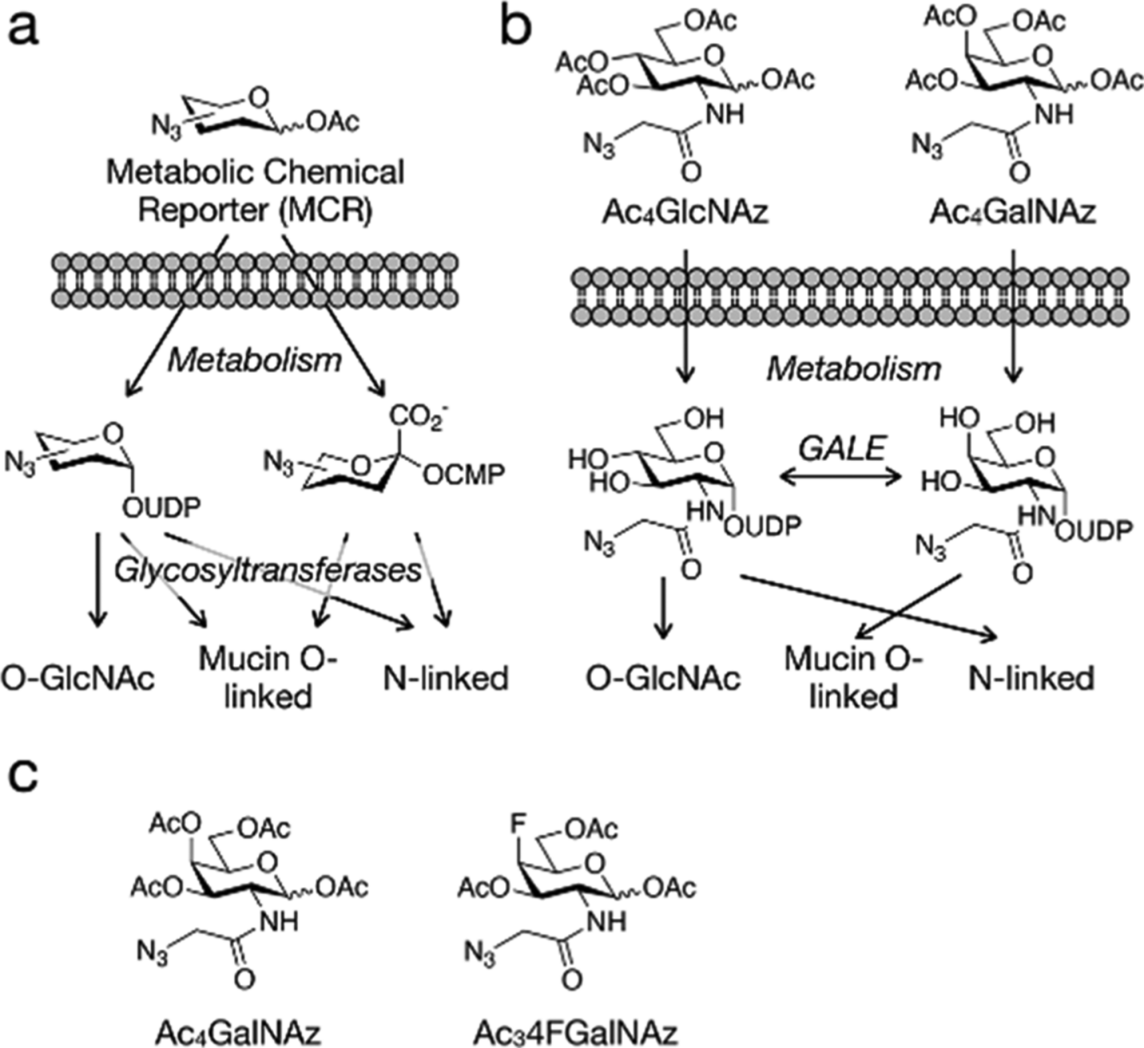
MCRs of glycosylation. (a) MCRs are monosaccharide analogues with bio-orthogonal functionalities. Cellular metabolism transforms MCRs into donor sugars where they are used by glycosyltransferases to modify glycoproteins. (b) GlcNAc- and GalNAc-based MCRs are typically nonselective, due in part to epimerization by the enzyme GALE. (c) Two reporters, Ac4Gal.

Metabolic chemical reporters (MCRs) of protein glycosylation are powerful chemical tools that have been used for over a decade to identify and characterize different types of glycans (Figure 1a).^1,2^ MCRs are typically analogues of naturally occurring monosaccharides that contain bio-orthogonal functionalities at different positions of the sugar ring. If these chemical modifications are relatively small, MCRs can take advantage of carbohydrate salvage pathway enzymes with different levels of substrate tolerance to yield the corresponding nucleotide sugar donors for subsequent transfer onto proteins by glycosyltransferases. Then, a second bio-orthogonal-chemistry step can be exploited for the selective installation of visualization and/or affinity tags.^3,4^ During the initial characterization of MCRs in the late 90s and early 00s, most of these probes were assumed to largely label one class of glycosylation. For example, Ac_4_GlcNAz was originally thought to label intracellular O-GlcNAc modifications, while its C4 epimer, Ac_4_GalNAz, seemed to largely label mucin O-linked glycosylation on the cell surface (Figure 1b).^5,6^ However, more careful analysis demonstrated that these two MCRs could be interconverted by the enzyme UDP-glucose 4-epimerase (GALE) after they reach their UDP donor sugars (Figure 1b).^7^ Therefore, treatment with either of these MCRs results in a mixture of labeled glycoproteins, which could complicate their clean application in certain types of experiments. This observation catalyzed an interest in the development of MCRs that are selective for one type of glycosylation over another. We and others have been the most successful in the creation of MCRs that are selective for O-GlcNAc modification, owing largely to what appears to be a fairly large substrate tolerance by O-GlcNAc transferase (OGT). For example, building upon previous *in vitro* observations with OGT, we demonstrated that both GlcNAc and glucose modified at the 6-position (e.g., 6-azido-6-deoxy-GlcNAc or 6AzGlcNAc) were selective reporters of O-GlcNAc.^8−10^ Independently, we and the Vocadlo lab also found that 2-azido-2-deoxy-glucose (2AzGlc) selectively labeled the O-GlcNAc glycome.^11,12^ Finally, work by Wang and co-workers demonstrated that 4-deoxy-GlcNAz was also a selective reporter of O-GlcNAc modifications.^13^

**Figure 2:**
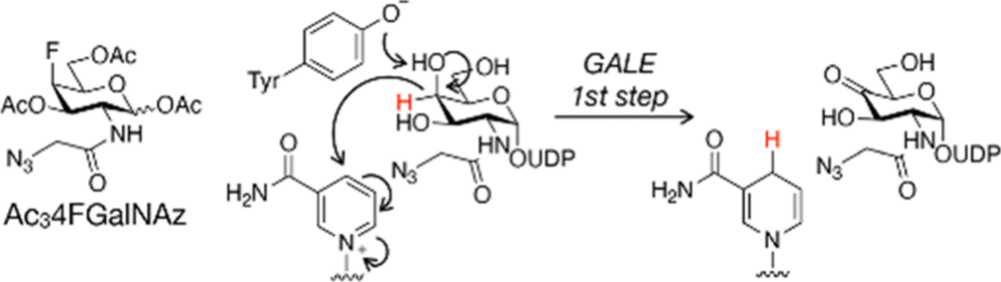
Design of Ac_3_4FGalNAz. The axial fluorine of 4FGalNAz cannot participate in the hydride abstraction reaction critical to UDP-GlcNAc/GalNAc epimerization by GALE.

Unfortunately, the development of selective MCRs for mucin O-linked glycosylation has been more difficult. Much of the success in this area has built upon our preliminary observation that larger substitutions at the N-acetyl position of UDP-GlcNAc and UDP-GalNAc appeared to inhibit their interconversion by GALE.^14^ This was confirmed through a series of careful experiments by the Bertozzi and Schumann (a co-author here) labs to create GalNAc analogues that were not accepted by GALE and were therefore selective for cell-surface glycosylation and even glycosyltransferase-specific mucin O-linked glycosylation through a bump-hole strategy.^15,16^ While these first GalNAc-selective MCRs are powerful tools, the large N-acetyl groups limit their metabolism by the endogenous GalNAc salvage-pathway enzymes and therefore require their administration as protected 1-phosphate derivatives and engineering of the downstream enzyme AGX1, which is responsible for the generation of the corresponding UDP derivatives.^17^ We hypothesized that this limitation could be overcome through rational design of a new MCR, termed Ac_3_4FGalNAz, that contained the small α-azido-acetate of GalNAz and a 4-deoxy-4-fluoro modification (Figure 1c). Importantly, fluorine has been used often as a bioisostere for hydroxyl groups in carbohydrates.^18^ As mentioned above, GalNAz is accepted by the endogenous salvage pathway and metabolized to UDP-GalNAz. Importantly, 4-deoxy-4-fluoro-GalNAc also transits the salvage enzymes. Unfortunately, the resulting UDP-4FGalNAc potently feedback inhibits the production of endogenous UDP-GlcNAc/GalNAc presumably through the hexosamine biosynthetic enzyme glutamine fructose-6-phosphate amidotransferase (GFAT).^19−22^ However, we have shown that azido-substitution of the N-acetyl position of different UDP sugar blocks this feedback mechanism.^23^ With these data in mind, we reasoned that Ac_3_4FGalNAz could be converted to UDP-4FGalNAz by endogenous enzymes and be incompatible with epimerization by GALE (Figure 2), preventing the formation of the GlcNAc epimer. Several studies have found that OGT can transfer UDP-GalNAc to peptide substrates but at significantly lower efficiency compared to UDP-GlcNAc.^24−26^ Therefore, we hypothesized that UDP-4FGalNAz would be more compatible with the GALNT (ppGalNAc-T) family of enzymes that initiate mucin O-linked glycosylation, making Ac_3_4FGalNAz a selective mucin O-linked MCR.

Here, we describe the synthesis and characterization of Ac_3_4FGalNAz as an MCR. Using living cells, we found that Ac_3_4FGalNAz treatment results in protein and cell-surface labeling but at a reduced efficiency compared to Ac_4_GalNAz and that this labeling is O- or S-linked in nature. Importantly, we also found that Ac_3_4FGalNAz does not result in feedback inhibition of O-GlcNAc or glycosaminoglycan (GAG) modifications. Subsequent proteomics experiments unambiguously identified 4FGalNAz as a modification on mostly intracellular proteins that are known targets of OGT, while analogous treatment with Ac_4_GalNAz yielded the expected mixture of cell-surface and intracellular glycoproteins. This surprising result prompted us to explore whether UDP-4FGalNAz may be a substrate for OGT. Toward this goal, we performed a series of *in vitro* enzymatic experiments demonstrating that 4FGalNAz can transit through the enzymes of the salvage pathway and is indeed a substrate for OGT that outperforms UDP-GalNAc. In contrast, we observed essentially no turnover of UDP-4FGalNAz by GALNT1 or T2. Finally, we confirmed these results in living cells by showing that an inhibitor of OGT dramatically reduced protein labeling upon Ac_3_4FGalNAz treatment. While these results show that our initial design rationale for a mucin O-linked MCR turned out to be flawed, they also further confirm the surprising enzymatic flexibility of OGT for accepting xenobiotic monosaccharides.

## Results and Discussion

We first synthesized Ac_3_4FGalNAz over nine steps (Figure S1). First, we protected the anomeric position of GlcNAc (**1**) as an α-*O*-benzyl glycoside **2**, which we then further elaborated to yield 3,4-benzylidene **3** in good yields. We then reacted compound **3** with benzylbromide to give the fully protected monosaccharide **4**, which was subjected to reductive benzylidene opening, isolating the 4-hydroxyl group and yielding derivative **5**. We then activated the 4-hydroxyl with trifluoromethanesulfonic anhydride and inverted the resulting intermediate by reaction with tetrabutylammonium fluoride, resulting in *O*-benzyl-protected 4FGalNAc (**6**). We then removed the benzyl-protecting groups with hydrogenation to give **7**, followed by the removal of the *N*-acetate under acidic conditions to yield the free amino sugar **8**. Finally, we added the azidoacetic acid group to give 4FGalNAz (**9**) and then acetylated the hydroxyl groups, resulting in Ac_3_4FGalNAz.

**Figure 3:**
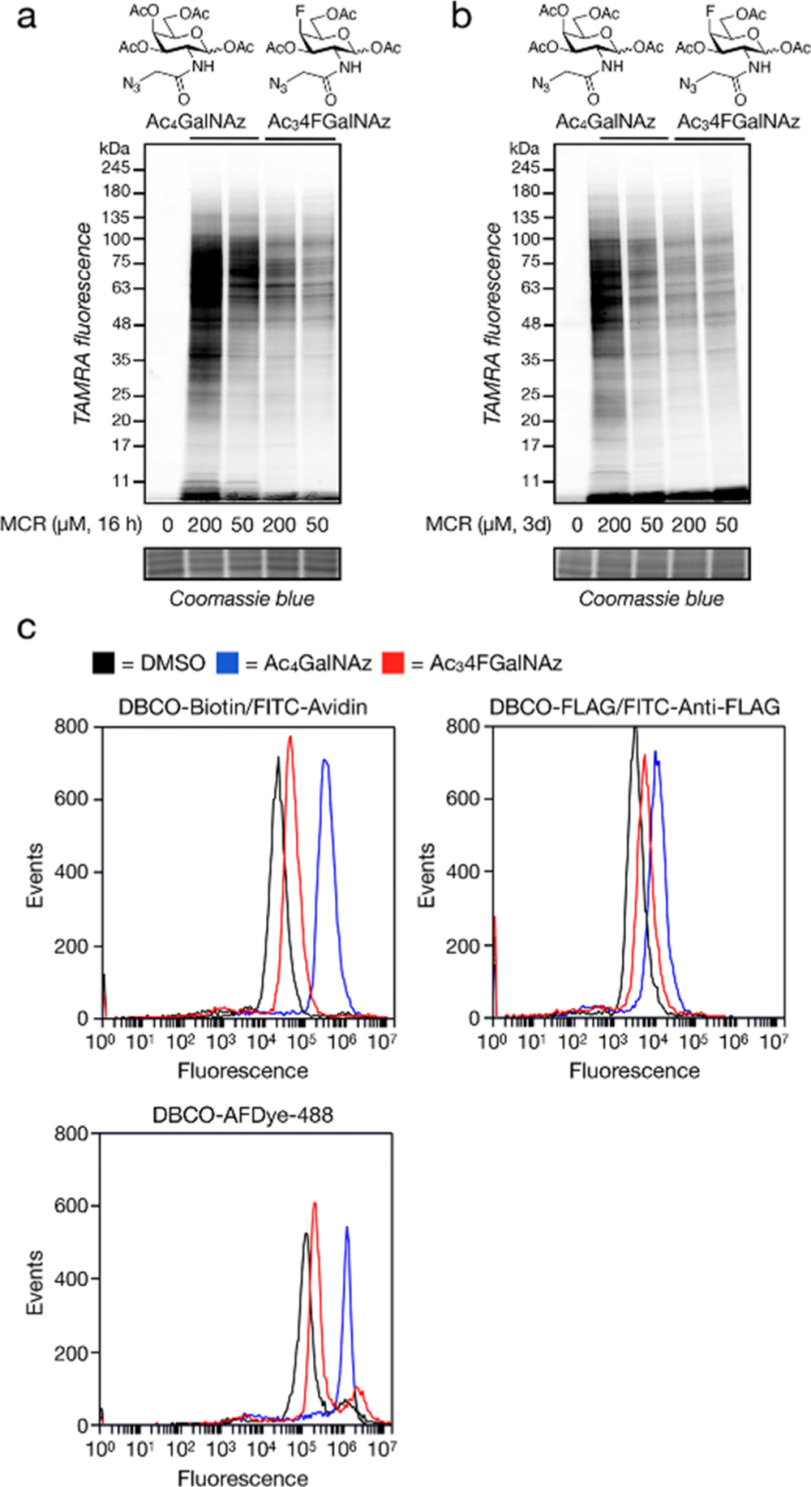
Ac_3_4FGalNAz treatment results in protein labeling in live cells. (a,b) 4FGalNAz labeling can be detected by in-gel fluorescence. CHO cells were treated with Ac_4_GalNAz or Ac_3_4FGalNAz (panel a: 16 h; panel b: 3 d) before CuAAC with TAMRA-alkyne and analysis by in-gel fluorescence. (c) 4FGalNAz labeling can be detected by flow cytometry. CHO cells were treated with individual MCRs (50 μM) for 3 d before the live cells were subjected to SPACC with the indicated DBCO regents and detection of fluorescence by flow cytometry.

With Ac_3_4FGalNAz in hand, we next set out to determine if it would label proteins in mammalian cells by treating CHO cells with either Ac_4_GalNAz or Ac_3_4FGalNAz at 50 or 200 μM for either 16 h (Figure 3a) or 3 d (Figure 3b). We chose CHO cells for this initial experiment as they have previously been used for GalNAz characterization.^6^ We subjected the corresponding cell lysates to CuAAC with alkyne-TAMRA and analyzed them by in-gel fluorescence scanning. As expected from published experiments, we observed robust labeling of a variety of proteins by GalNAz and gratifyingly reduced but notable labeling by 4FGalNAz. Next, we tested whether at least some of this labeling was localized to the cell surface. Specifically, we first treated CHO cells with 50 μM either Ac_4_GalNAz or Ac_3_4FGalNAz for 3 d. We then collected the live cells by gentle centrifugation and reacted any cell-surface azides using three different strain-promoted azide–alkyne cycloaddition reagents: DBCO-biotin followed by FITC-avidin, DBCO-FLAG followed by FITC-anti-FLAG antibody, or DBCO-AFDye-488. Using flow cytometry, we observed cell-surface labeling under all three methods for both GalNAz and 4FGalNAz, with 4FGalNAz again showing a low signal (Figure 3c).

**Figure 4:**
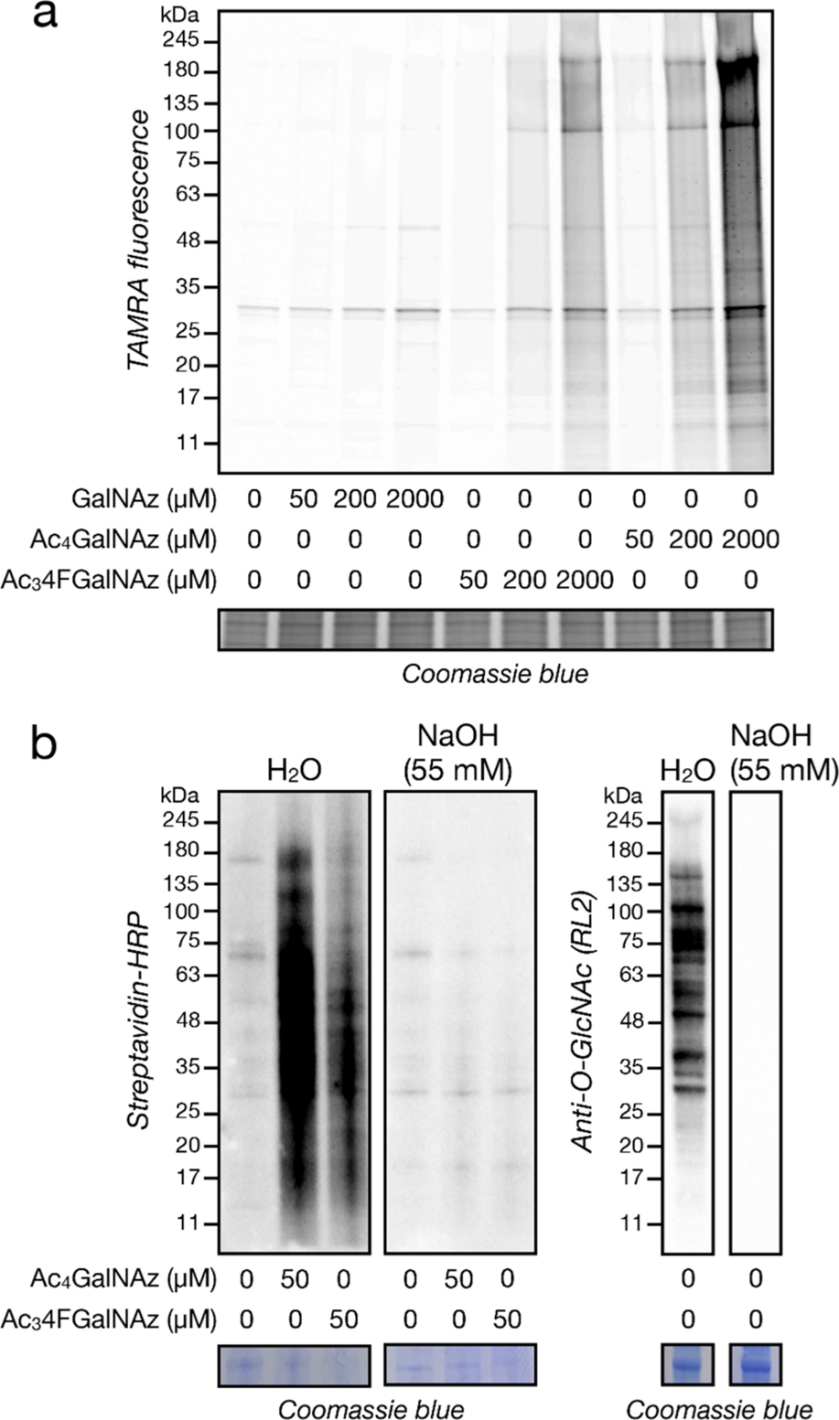
Ac_3_4FGalNAz modifies proteins through largely an O-linkage. (a) 4FGalNAz displays relatively reduced background chemical-labeling of cysteines. The indicated concentrations of various MCRs were incubated with cell lysates before CuAAC with TAMRA-alkyne and in-gel fluorescence. (b) β-Elimination removes a 4FGalNAz signal. CHO cells were treated with the individual MCRs (50 μM) for 3 d before CuAAC with biotin-alkyne and visualization by streptavidin blot. β-Elimination (55 mM NaOH) removes this signal. Anti-O-GlcNAc western blotting is a positive control.

Careful examination of per-O-acetylated monosaccharide MCRs by the Chen lab uncovered background chemical modification of protein cysteines that might obscure a glycosyltransferase-mediated signal under certain circumstances.^27^ More specifically, they found that deacetylation of the anomeric position can be followed by elimination of the 3-*O*-acetate, resulting in the formation of a Michael acceptor that reacts with nucleophilic cysteine residues.^28^ To test if the Ac_3_4FGalNAz might also result from this type of chemical modification, we followed the Chen lab protocol and incubated native cell lysates with a range of concentrations (50–2000 μM) of GalNAz, Ac_4_GalNAz, or Ac_3_4FGalNAz. Consistent with the Chen lab results, we observed significant protein labeling by Ac_4_GalNAz at higher concentration and that the majority of this signal was absent in free GalNAz (Figure 4a). We found that Ac_3_4FGalNAz displays an intermediate level of lysate labeling, with essentially no background reactivity at our chosen concentration of 50 μM for cell-based experiments. Finally, we set out to determine if Ac_3_4FGalNAz protein labeling was mostly O(S)- or N-linked to proteins by again treating CHO cells with 50 μM either Ac_4_GalNAz or Ac_3_4FGalNAz for 3 d. We then performed CuAAC with alkyne-biotin, separated the proteins by sodium dodecyl sulphate–polyacrylamide gel electrophoresis (SDS–PAGE), and transferred them in duplicate to a polyvinylidene fluoride (PVDF) membrane. We subjected the membranes to either H_2_O (as a control) or NaOH (55 mM) at 50 °C overnight, which results in the β-elimination of O- and S-linked glycans. Upon blotting with HRP-linked streptavidin, we observed loss of essentially all labeling corresponding to both the Ac_4_GalNAz and Ac_3_4FGalNAz (Figure 4b). Taken together, these data demonstrate that mammalian cell proteins are indeed labeled upon Ac_3_4FGalNAz treatment, most likely through O-linkages.

**Figure 5:**
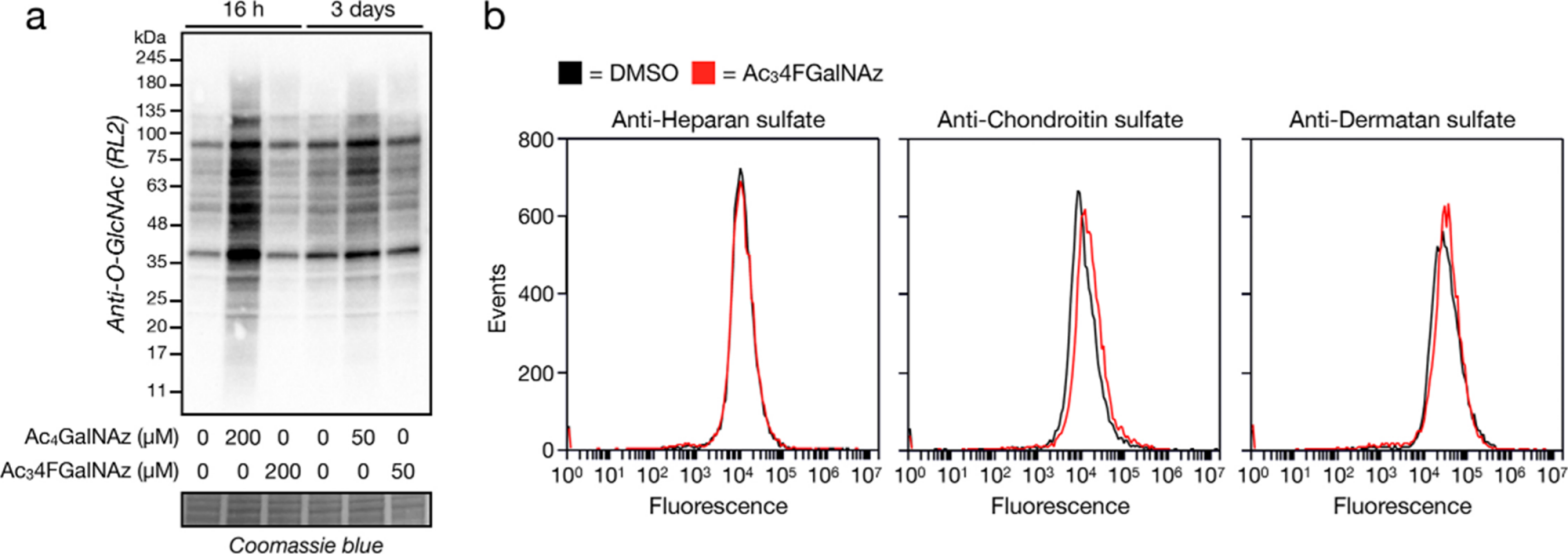
Ac_3_4FGalNAz treatment does not inhibit O-GlcNAc or GAGs. (a) 4FGalNAz does not inhibit O-GlcNAc modifications. CHO cells were treated under the indicated conditions before visualization of O-GlcNAc levels by western blotting. (b) 4FGalNAz does not inhibit GAGs. CHO cells were treated with Ac_3_4FGalNAz (50 μM) for 3 days before the live cells were analyzed using GAG-specific antibodies by flow cytometry.

Treatment of cells with Ac_3_4FGalNAc results in inhibition of both O-GlcNAc modifications and GAGs on the cell surface by reducing the cellular concentrations of UDP-GlcNAc and UDP-GalNAc, presumably through its conversion to UDP-4FGalNAc and feedback inhibition of the biosynthesis of UDP-GlcNAc by GFAT.^22^ To test if 4FGalNAz might affect O-GlcNAc modifications, we again treated CHO cells with either Ac_4_GalNAz or Ac_3_4FGalNAz (200 μM, 16 h or 50 μM for 3 d) and performed western blotting using an anti-O-GlcNAc antibody (Figure 5a). In the case of Ac_4_GalNAz, we observed an increase in antibody staining, which we reasoned could result from detection of the resulting GlcNAz moieties by the RL2 antibody. Each pan O-GlcNAc antibody has different underlying protein preferences, so we do not know if the same increase would be observed with other antibodies. Importantly, we did not find any inhibition of O-GlcNAc upon Ac_3_4FGalNAz treatment. We then treated CHO cells (50 μM, 3 d) with Ac_3_4FGalNAz and detected heparan sulfate, chondroitin sulfate, or dermatan sulfate using flow cytometry (Figure 5b). Once again, we detected no loss of any GAG chains upon Ac_3_4FGalNAz treatment. These results demonstrate that 4FGalNAz does not have the same detrimental effect as 4FGalNAc on endogenous glycosylation. They are consistent with our published observation that increased steric bulk at the N-acetyl position of UDP-GlcNAc or UDP-GalNAc, such as an azide, prevented feedback inhibition of GFAT.^23^ Finally, we used an MTT assay to show that Ac_3_4FGalNAz was not toxic to cells at 50 μM and displayed a similar toxicity to Ac_4_GalNAz at 200 μM (Figure S1).

**Figure 6:**
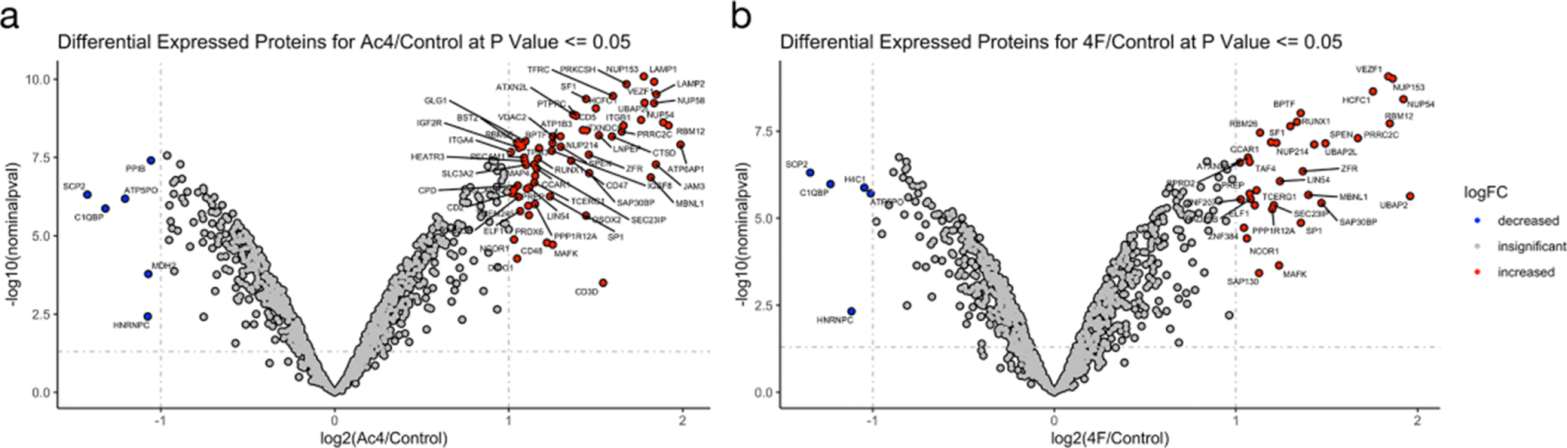
Proteomic analysis of MCR-labeled proteins. Jurkat cells were treated with (a) Ac_4_GalNAz (50 μM) or (b) Ac_3_4FGalNAz (50 μM) for 3 d. Labeled proteins were then enriched using neutravidin beads after CuAAC with IsoTaG alkyne-biotin. Proteins were then identified using label-free quantitation after on-bead trypsin digestion and LC–MS/MS. The results are shown as a volcano plot (*x*-axis: log 2 ratio of MCR to DMSO vehicle, *y*-axis; −log 10 *p*-value). Significantly enriched proteins that differ at least 2 linearfold with a *p*-value < 0.05 (Student’s *t*-test) are marked in red.

With these initial characterization experiments completed, we moved on to perform glycoproteomics. Specifically, we employed the IsoTaG platform to identify specific modification sites and glycans labeled by GalNAz and/or 4FGalNAz.^29,30^ We chose to perform the experiment in Jurkat cells as they have fairly simple mucin O-linked glycans due to a mutant COSMC chaperone.^31^ First, we treated Jurkat cells with either Ac4GalNAz (50 μM), Ac_3_4FGalNAz (50 μM), or DMSO vehicle for 3 d and found that 4FGalNAz does not inhibit O-GlcNAc modification (Figure S3a) and results in cell-surface labeling at similar levels compared to CHO cells (Figure S3b). We then repeated these treatment conditions and performed CuAAC on the corresponding lysates with a mixture of isotopically labeled, cleavable biotin tags and selectively enriched the labeled proteins. Next, we used Byonic and IsoStamp v2.0 software to assign peptides containing either GalNAz or 4FGalNAz modifications, as well as more elaborated glycan structures. With IsoTaG, we identified 67 GalNAz-modified proteins significantly enriched over the DMSO control (greater than or equal to twofold change; *p*-value < 0.05; Figure 6a, Table S1), and we localized GalNAz to 147 unique peptides (122 sites at S/T and 29 sites at C after filtering for peptide spectral matches ≥2; corresponds to 34 unambiguous glycosites identified by EThcD and delta Mod ≥10, Tables S2 and S3), representing the expected mixture of cell-surface and intracellular glycoproteins. Consistent with its overall lower levels of labeling, we identified fewer enriched (36) 4FGalNAz proteins (Figure 6b, Table S1), as well as site identifications (97 unique peptides; 91 sites at S/T and 6 sites at C after filtering for peptide spectral matches ≥ 2; corresponds to 19 unambiguous glycosites identified by EThcD and delta Mod ≥ 10, Tables S2 and S3). In contrast to our hypothesis that 4FGalNAz would be a more selective reporter for mucin O-linked glycosylation, we found that almost all of the 4FGalNAz-modified proteins were intracellular and many were known to be O-GlcNAcylated (e.g., HCF-1 and NUP153).

**Figure 7:**
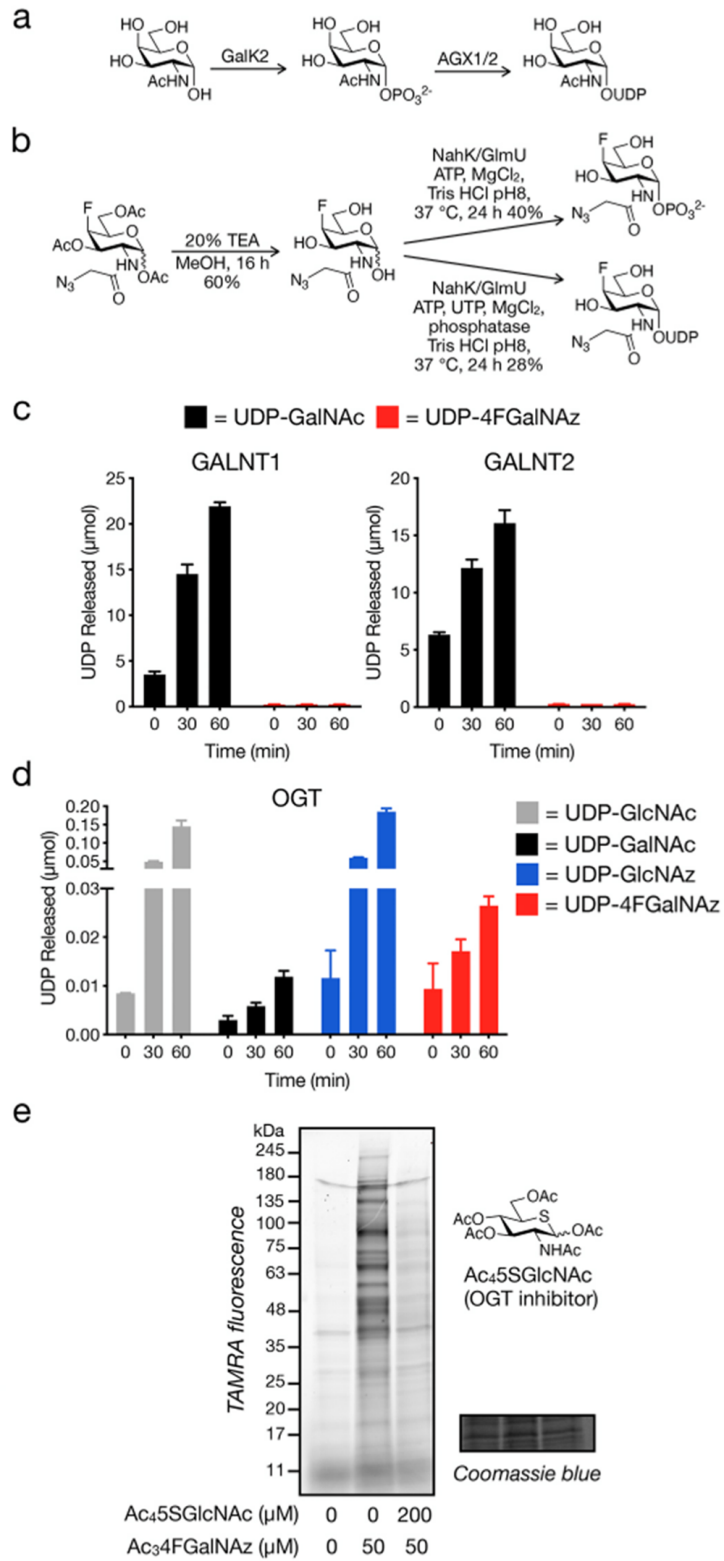
4FGalNAz is an O-GlcNAc reporter. (a) GalNAc-salvage pathway of mammalian cells. (b) Chemoenzymatic synthesis of 4FGalNAz-1-phosphate and UDP-4FGalNAz. (c) *In vitro* GALNT activity with various nucleotide sugars. A luminescence-based coupled enzyme assay (UDP-Glo; Promega) utilizing UDP-GalNAc or UDP-4FGalNAz at 50 and 50 μM peptide substrate was used to assess GALNT1 and GALNT2 activity. Data represent the mean, and error bars represent a standard deviation of three trials. (d) *In vitro* ncOGT activity with various nucleotide sugars. A luminescence-based coupled enzyme assay (UDP-Glo; Promega), utilizing UDP-GlcNAc, UDP-GalNAc, UDP-GlcNAz, and UDP-4F-GalNAz all at 40 and 125 μM peptide substrate, was used to assess ncOGT activity. Data represent the mean, and error bars represent a standard deviation of three trials.

To investigate this somewhat unexpected result, we next set out to characterize the ability of different enzymes to utilize 4FGalNAz and its associated metabolites *in vitro*. As we mentioned in the introduction, GalNAz-based MCRs are thought to be biosynthesized into UDP sugar donors by the enzymes of the GalNAc-salvage pathways (Figure 7a). Briefly, GalNAc is first phosphorylated at the anomeric position by GalK2, followed by conjugation with UTP to form UDP-GalNAc by AGX1 (or UAP1). UDP-GalNAc can then be used by glycosyltransferases, including the GALNT (ppGalNAcT) family. To test if 4FGalNAz is a substrate for these enzymes, we first prepared the relevant substrates using a chemoenzymatic strategy (Figure 7b). First, we removed the acetates from Ac_3_4FGalNAz to yield the associated free sugar. We then subjected 4FGalNAz to enzymatic transformation using the fused version of two bacterial enzymes, an N-acetylhexosamine kinase (NahK) and a GlcNAc-1-P uridyltransferase (GlmU).^32^ To generate UDP-4FGalNAz, we added both ATP and UTP and obtained 4FGalNAz-1-phosphate by omitting the UTP. With these metabolites in hand, we then attempted to obtain Michaelis–Menten kinetic constants using recombinant GalK2 and AGX1. We found that the enzymes were able to turn over 4FGalNAz and 4FGalNAz-1-phosphate, respectively, albeit with reduced efficiency compared to the natural GalNAc substrates (Figure S2). We next tested GALNT1 and GALNT2 with UDP-GalNAc or UDP-4FGalNAz (50 μM) and the standard peptide acceptor MUC5AC using the UDP-Glo assay from Promega (Figure 7c). Consistent with our proteomics data, we could not detect any GALNT activity with UDP-4FGalNAz despite clear turnover of the native UDP-GalNAc substrate. Next, we used the same UDP-Glo assay to test OGT activity against a small panel of UDP donor sugars (Figure 7d). We confirmed previously published data showing that OGT accepted UDP-GalNAc but less efficiently than UDP-GlcNAc and that the MCR UDP-GlcNAz was a good OGT substrate.^24−26^ We also found that UDP-4FGalNAz was also a substrate accepted about 2.5 times better than UDP-GalNAc but less efficiently than UDP-GlcNAc or UDP-GlcNAz. Finally, we set out to confirm whether the 4FGalNAz signal we observed in cells resulted from OGT activity. Accordingly, we pretreated CHO cells with the OGT inhibitor Ac_4_5SGlcNAc (200 μM) for 24 h before the addition of Ac_3_4FGalNAz (50 μM) for an additional 24 h. Using in-gel fluorescence, we found that OGT-inhibitor treatment caused a major reduction in the fluorescent signal (Figure 7e), confirming that the majority of 4FGalNAz labeling is indeed due to OGT activity.

## Conclusions

MCRs are powerful tools for the labeling and subsequent visualization/identification of glycoproteins. Since their introduction, we and others have created MCRs built on several monosaccharide scaffolds including GlcNAc, GalNAc, ManNAc, sialic acid, and fucose.^1,2^ Unfortunately, several of these monosaccharides can be interconverted by cellular metabolism, rendering the corresponding MCRs nonselective for different classes of glycans. This has been a particularly challenging problem for GlcNAc-based and GalNAc-based MCRs due to reversible epimerization of UDP-GlcNAc and UDP-GalNAc by GALE. We have had some success at creating GalNAc selective reporters by building on the fact that large N-acetyl substituents render UDP-GalNAc refractory to epimerization by GALE.^14−16^ However, these same large modifications require engineering of enzymes in the GalNAc-salvage pathway to ensure their metabolism. Here, we attempted to overcome the requirement for biosynthetic-pathway engineering through the synthesis and evaluation of 4FGalNAz. We hypothesized that the axial fluorine would act as an isostere for the electronics of the 4-hydroxyl group of GalNAz and be impossible to epimerize to the GlcNAc stereochemistry by GALE. Therefore, we reasoned that the stereochemistry of 4FGalNAz would allow it to be efficiently accepted by the mucin GALNT glycosyltransferases over OGT. Our hypothesis initially seemed reasonable as treatment of cells with Ac_3_4FGalNAz resulted in protein labeling and some cell-surface signals that could be detected by flow cytometry, albeit much less than upon Ac_4_GalNAz treatment (Figure 3). However, we used subsequent proteomics, *in vitro* biochemistry, and competition with an OGT inhibitor in cells to discover that UDP-4FGalNAz is not a detectable substrate for GALNT1 or 2 but is accepted by OGT (Figures 6 and 7). We do not know the underlying origins of the positive flow cytometry signal is coming from but believe that it could arise from acceptance of UDP-4FGalNAz by other glycosyltransferases that generate oligosaccharide branches. Interestingly, Ac_3_4FGalNAz yields less “background” chemical labeling of proteins in lysates compared to Ac_4_GalNAz (Figure 4) characterized by the Chen lab.^28^ In this process, lysine residues act as a base for a β-elimination reaction to generate a Michael acceptor that is then trapped by cysteine residues. One would predict that the strong electron-withdrawing character of fluorine would increase the rate of the elimination reaction. Therefore, the exact reason for the lower levels of background modification is mysterious. We believe it may result from reduced noncovalent interactions between Ac_3_4FGalNAz and the proteins in the lysate, resulting in fewer opportunities for lysine residues to catalyze the elimination. Finally, we found that Ac_3_4FGalNAz does not inhibit O-GlcNAc or GAG modifications (Figure 5) unlike Ac_3_4FGlcNAc.^22^ As mentioned above, this result matches well with our previous *in vitro* analysis where we demonstrated that modifications on the N-acetyl position of UDP-GalNAc prevent feedback inhibition of GFAT.^23^ Notably, Ac_4_GalNAz treatment resulted in increased O-GlcNAc modification as detected by the anti-O-GlcNAc antibody RL2 (Figure 5a). We attribute this to likely recognition of O-GlcNAz by RL2.

Taken together, our results are obviously disappointing given our initial goal of creating an MCR for the core of mucin O-linked glycosylation. However, they do have interesting implications for OGT biology. First, our discoveries once again highlight the promiscuity of OGT for a variety of UDP sugar donors, a list that includes the native sugars GlcNAc, GalNAc,^24−26^ and glucose,^12^ as well as 2-azido-glucose,^11,12^ 6-azido- and 6-alkynyl-GlcNAc,^8,9^ 4-deoxy-GlcNAz,^13^ and 6-azido-glucose.^10^ It is unclear why OGT has avoided evolutionary pressure to only transfer GlcNAc, and our results suggest that it will be difficult to simply “dial-out” OGT activity as a strategy to generate selective MCRs for other types of glycosylation. Furthermore, UDP-4FGalNAz is only accepted by OGT approximately twice as well as UDP-GalNAc but results in notable protein labeling, suggesting that O-GalNAc placed by OGT may be a more common modification than previously appreciated. It may also explain some of the nuclear O-GalNAc that has been detected but attributed to GALNT3.^33^

## Methods

### Cell Culture

CHO cells (ATCC) were cultured in Ham’s F12K media (Genesee Scientific) enriched with 10% fetal bovine serum (FBS, Atlanta Biologics). Jurkat cells (ATCC) were cultured in RPMI (Genesee Scientific) enriched with 10% FBS. All cell lines were incubated at 37 °C with 5.0% CO_2_ in a humidified incubator.

### Metabolic Labeling

To cells at 80–85% confluency, media containing Ac_4_GalNAz, Ac_3_4FGalNAz (1000× stock in DMSO), or DMSO vehicle was added as indicated for 16 h. For longer treatment (3 days), media with Ac_4_GalNAz, Ac_3_4FGalNAz (1000× stock in DMSO), or DMSO vehicle was added to cells at 20–25% confluency as indicated.

### Analysis by In-Gel Fluorescence

Cells were collected via scraping in phosphate-buffered saline (PBS) and pelleted by centrifugation for 4 min at 2000*g* at 4 °C. Cells were resuspended in 4% SDS buffer (4% SDS, 150 mM NaCl, 50 mM TEA pH 7.4) with cOmplete, mini, EDTA-free protease inhibitor cocktail tablets (Roche, 5 mg mL^–1^) and tip-sonicated at 35% amplitude for 20, 5 s on 5 s off, and centrifuged for 10 min at 10,000*g*. The supernatant was collected, and protein concentration was determined by BCA assay. Protein concentration was normalized to 1 μg/μL. To 200 μg of protein normalized to 1% SDS, 12 μL of freshly made click chemistry cocktail was added and gently vortexed and allowed to sit at room temperature in the dark for 1 h [Alkyne-TAMRA tag (Click Chemistry tools, 100 μM, 10 mM stock solution in DMSO); tris(2-carboxyethyl)phosphine hydrochloride (TCEP) (1, 50 mM freshly prepared stock solution in water); tris[(1-benzyl-1*H*-1,2,3-triazol-4-yl)methyl]amine (TBTA) (100 μM, 10 mM stock solution in DMSO); and CuSO_4_·5H_2_O (1, 50 mM freshly prepared stock solution in water)]. Proteins were precipitated using ice-cold methanol and placed at −20 °C for at least 2 h before being spun down (10 min, 10,000*g* at 4 °C). The supernatant was poured off, the protein pellet was allowed to air-dry for 5–10 min before 50 μL of 4% SDS buffer was added, and the samples were bath-sonicated for complete dissolution. To the samples, 50 μL of SDS-free 2× loading buffer (100 mM Tris, 20% glycerol, 0.2% bromophenol blue, and 1.4% β-mercaptoethanol, pH 6.8) was added and then boiled at 95 °C for 5 min and 40 μg was loaded per lane for SDS–PAGE (Criterion TGX 4–20% Gel, Bio-Rad) separation. Following separation, gels were scanned on a Typhoon 9400 variable mode imager (GE Healthcare) using 532 nm for excitation and a 30 nm band-pass filter centered at 610 nm for detection.

### Cell-Surface Labeling by Flow Cytometry with DBCO-Biotin

CHO cells were treated at 20–25% confluency with Ac_4_GalNAz, Ac_3_4FGalNAz, or DMSO for 3 days in triplicate. Cells were collected using 10 mM EDTA in PBS (pH 7.4) for 10 min at 37 °C after gently washing cells in PBS. Cells were pelleted (5 min, 800*g* at 4 °C) and washed three times with ice-cold PBS. Cells were resuspended in 200 μL of 60 μM DBCO-biotin in PBS and allowed to react for 1 h at room temperature before pelleting and washing three times with ice-cold PBS as previously stated. The pellet was resuspended in ice-cold PBS containing avidin-conjugated fluorescein isothiocynate (FITC) at 5 μg mL^–1^ (Sigma) and incubated on ice for 30 min before pelleting and washing three times with ice-cold PBS. Cells were resuspended in 500 μL of PBS containing propidium iodide (2.5 μg mL^–1^) for 30 min for dead cell exclusion and then 10,000 cells were analyzed on a BD SORP LSRII flow cytometer using the 488 nm argon laser.

### Cell-Surface Labeling by Flow Cytometry with DBCO-FLAG

CHO cells were treated at 20–25% confluency with Ac_4_GalNAz, Ac_3_4FGalNAz, or DMSO for 3 days in triplicate at which time cells were harvested as previously described using 10 mM EDTA in PBS (pH 7.4), pelleted, and washed with ice-cold 1% FBS in PBS three times (5 min, 800*g* at 4 °C). Cells were resuspended in 250 μM DBCO-PEG_4_-FLAG (Jena Bioscience) in 1% FBS in PBS and incubated for 1 h at room temperature and subsequently washed three times in ice-cold 1% FBS in PBS. The cell pellet was resuspended in FITC-anti-FLAG (Sigma) diluted 1:900 in 1% FBS in PBS for 30 min on ice. Cells were pelleted and washed three times before resuspension in 500 μL of 1% FBS in PBS containing 2.5 μg mL^–1^ propidium iodide for dead cell exclusion and 10,000 cells were analyzed.

### Cell-Surface Labeling by Flow Cytometry with AFDye 488 DBCO

CHO cells were treated at 20–25% confluency with Ac_4_GalNAz, Ac_3_4FGalNAz, or DMSO in triplicate for 3 days before collection via cell dissociation buffer enzyme-free PBS-based (Gibco) for 10 min at 37 °C and pelleting via centrifugation (5 min, 800*g* at 4 °C). Cells were washed three times with ice-cold 1% FBS in PBS before resuspension in 50 μM AFDye 488 DBCO (Click Chemistry Tools) for 1 h at room temperature. Cells were pelleted and washed three times as previously stated and resuspended in 500 μL of 1% FBS in PBS. Dead cells were excluded using 2.5 μg mL^–1^ propidium iodide and 10,000 cells were analyzed.

### Detection of Cell-Surface GAGs by Flow Cytometry

CHO cells were treated at 20–25% confluency with Ac_4_GalNAz, Ac_3_4FGalNAz, or DMSO in triplicate for 3 days at which time cells were collected using cell dissociation buffer enzyme-free PBS-based (Gibco) for 10 min at 37 °C and collected by centrifugation (5 min, 1000*g* at 4 °C). Cells were fixed in 4% paraformaldehyde in PBS on ice for 10 min and pelleted (5 min, 1000*g* at 4 °C) before being washed twice in ice-cold PBS. Primary antibodies (HS4C3 at 1:20; IO3H10 at 1:10; GD3A12 at 1:10)^21,22^ were diluted in FACS buffer (0.2% BSA in PBS) and incubated with cells for 1 h at 4 °C. Cells were then washed two times with ice-cold PBS before incubation with anti-VSV (P5D4) at 1:10 dilution in FACS buffer for 45 min at 4 °C and then washed twice in ice-cold PBS. Goat anti-mouse IgG (H + L) and Alexa Fluor 488 conjugate antibody (Sigma) were diluted 1:500 in FACS buffer and incubated with cells for 45 min at 4 °C. Cells were washed twice with ice-cold PBS before resuspension in 500 μL of PBS for flow cytometry analysis. A total of 10,000 cells were analyzed on a BD SORP LSRII flow cytometer using the 488 nm argon laser.

### RL2 Blotting

CHO cells were treated at 80–85% confluency for 16 h treatment or 20–25% confluency for 3 day treatment with Ac_4_GalNAz, Ac_3_4FGalNAz, or DMSO before collecting via scrapping in PBS and pelleting by centrifugation (4 min, 2000*g* at 4 °C). Cells were resuspended in 4% SDS lysis buffer supplemented with cOmplete, mini, EDTA-free protease inhibitor cocktail tablets before tip sonication on ice (20% amplitude, 15 s pulse, 5 s on 5 s off) and centrifugation (10 min, 10,000*g* at 4 °C). The supernatant was collected and protein concentration was determined using BCA assay and normalized to 2 mg mL^–1^ using SDS-free 2× loading buffer (100 mM Tris, 20% glycerol, 0.2% bromophenol blue, and 1.4% β-mercaptoethanol, pH 6.8). The mixtures were boiled at 95 °C for 5 min before loading 30 μg per lane for SDS–PAGE separation. Following separation, proteins were transferred to a PVDF membrane (Bio-Rad) using manufacturer’s protocols. The blot was then washed in TBST once for 10 min before blocking for 1 h at rt in OneBlock Western-CL blocking buffer (Genessee Scientific). The blot was then incubated at 4 °C for 16 h with anti-RL2 antibody (Thermo Scientific) at 1:5000 dilution in blocking buffer. The blot was washed three times with TBST for 5 min each before incubation with anti-mouse IgG for 1 h at rt at 1:10000 in blocking buffer. The blot was imaged using ECL reagents after washing with TBST three times for 5 min each.

### Beta-Elimination

CHO cells were treated at 20–25% confluency with Ac_4_GalNAz, Ac_3_4FGalNAz, or DMSO at 50 μM for 3 days before harvesting with cell dDissociation buffer enzyme-free PBS-based (Gibco) for 10 min at 37 °C and collected by centrifugation (4 min, 2000*g* at 4 °C) and washed twice with PBS. Cells were resuspended in 4% SDS lysis buffer (4% SDS, 150 mM NaCl, and 50 mM TEA pH 7.4) supplemented with cOmplete, mini, EDTA-free protease inhibitor cocktail tablets (Roche, 5 mg mL^–1^) and tip-sonicated on ice at 20% amplitude for 15 s pulse, 5 s on and 5 s off. The supernatant was collected after centrifugation (10 min, 10000*g* at 4 °C), and protein concentration was determined via BCA assay (Pierce, Thermo Scientific). Protein was either normalized to 2 mg mL^–1^, and to 100 μg freshly made click chemistry cocktail (7 μL) were added Alkyne-biotin tag (Click Chemistry tools, 100 μM, 10 mM stock solution in DMSO); tris(2-carboxyethyl)-phosphine hydrochloride (TCEP) (1, 50 mM freshly prepared stock solution in water); tris[(1-benzyl-1-H-1,2,3-triazol-4-yl)methyl]-amine (TBTA) (100 μM, 10 mM stock solution in DMSO); and CuSO_4_·5H_2_O (1, 50 mM freshly prepared stock solution in water) after normalization to 1% SDS using SDS-free buffer (10 mM TEA pH 7.4 and 150 mM NaCl) and 1.25% SDS buffer (2.5% SDS, 10 mM TEA pH 7.4, and 150 mM NaCl) for streptavidin horseradish peroxidase (Strep-HRP), or 100 μg of DMSO treatment was diluted to 2 mg mL^–1^ using SDS-free 2× loading buffer (100 mM Tris, 20% glycerol, 0.2% bromophenol blue, and 1.4% β-mercaptoethanol, pH 6.8) for RL2 analysis. Click reactions were gently vortexed and allowed to sit at rt for 1 h before the addition of 1 mL of ice-cold methanol and placement at −20 °C for 2 h for protein precipitation. The reaction mixtures were then centrifuged for 10 min at 10,000*g* at 4 °C, the supernatant poured off, and protein pellets were allowed to air-dry for 10 min. Protein was resuspended in 50 μL of 4% SDS buffer and gently sonicated in a bath sonicator and 50 μL of 2× SDS-free loading buffer was added to the mixture. The samples were boiled at 95 °C for 5 min before 5 μg of proteins for Strep-HRP analysis and 15 μg of protein for RL2 analysis were loaded per lane for separation via SDS–PAGE (Criterion TGX 4–20% Gel, Bio-Rad). Proteins were transferred to the PVDF membrane (Bio-Rad) using manufacturer’s protocols and then washed in TBST for 10 min one time. Blot was incubated either in H_2_O or 55 mM NaOH at 40 °C for 24 h. Blots were then washed with TBST 3 × 5 min each and blocked for 1 h at room temperature in OneBlock Western-CL blocking buffer (Genessee Scientific). RL2 analysis was incubated overnight at 4 °C with anti-RL2 diluted 1:5000 in blocking buffer, washed 3× in TBST, and then incubated for 1 h at rt with anti-mouse 1:10000 in blocking buffer. Strep-HRP analysis was incubated at rt for 1 h with Strep-HRP diluted 1:5000 in blocking buffer. Blots were washed with TBST 3 × 5 min each before imaging using ECL reagents.

### MTT Assay

CHO cells (2.5 × 10^4^ cells) were plated per well in a 96-well poly-d-lysine-coated dish for 24 h before treatment with DMSO, Ac_4_GalNAz, or Ac_3_4FGalNAz at 50 μM for 3 days. CellTiter 96 aqueous non-radioactive cell proliferation assay (Promega) was provided according to manufacturer’s protocol. Absorbance at 490 nm was read using a BioTek Synergy H4 multimode microplate reader.

### Glycoproteomics

#### Chemical Enrichment of Glycoproteins and Sample Preparation for IsoTag

Using established protocols essentially as described by Darabedian et al.,^10,34^ we used the following methods, which are included here for clarity and detail-specific variations. The cell pellets were lysed on ice by probe tip sonication in 1× PBS + 2% SDS (0.5 mL), containing EDTA-free Pierce HaltTM protease inhibitor cocktail. Debris were removed from the cellular lysate by centrifugation (20,000*g*) for 20 min at 4 °C, and the supernatant was transferred to a new Eppendorf tube. A BCA protein assay (Pierce) was performed, and protein concentration was adjusted to 7.5 μg/μL with lysis buffer. Protein lysate (3 mg, 400 μL) was treated with a premixed solution of the click chemistry reagents [100 μL; final concentration of 200 μM IsoTaG silane probe (3:1 heavy/light mixture), 500 μM CuSO_4_, 100 μM THPTA, 2.5 mM sodium ascorbate], and the reaction mixture was incubated for 3.5 h at 24 °C. The click reaction was quenched by methanol–chloroform protein precipitation [aqueous phase/methanol/chloroform = 4:4:1 (v/v/v)]. The protein pellet was allowed to air-dry for 5 min at 24 °C. The dried pellet was resuspended in 1× PBS + 1% SDS (400 μL) by probe tip sonication and then diluted in PBS (1.6 mL) to a final concentration of 0.2% SDS. Streptavidin-agarose resin [400 μL, washed with PBS (3 × 1 mL)] was added to the protein solution and the resulting mixture was incubated for 12 h at 24 °C with rotation. The beads were washed using spin columns with 8 M urea (5 × 1 mL) and PBS (5 × 1 mL). The washed beads were resuspended in 500 μL of PBS containing 10 mM DTT and incubated at 37 °C for 30 min, followed by the addition of 20 mM iodoacetamide for 30 min at 37 °C in the dark. The reduced and alkylated beads were collected by centrifugation (1500*g*) and resuspended in 520 μL of PBS. Urea (8 M, 32 μL) and trypsin (1.5 μg) were added to the resuspended beads, and digestion was performed for 16 h at 37 °C with rotation. The supernatant was collected, and the beads were washed three times with PBS (200 μL) and distilled water (2 × 200 μL). Washes were combined with the supernatant digest to form the trypsin fractions for protein identification. The IsoTaG silane probe was cleaved with 2% formic acid/water (2 × 200 μL) for 30 min at 24 °C with rotation and the eluent was collected. The beads were washed with 50% acetonitrile–water + 1% formic acid (2 × 500 μL), and the washes were combined with the eluent to form the cleavage fraction for site level identification. The trypsin and cleavage fractions were dried in a vacuum centrifuge and desalted using C18 tips following the manufacturer’s instructions. Trypsin fractions were resuspended in 50 mM TEAB (20 μL), and the corresponding amine-based TMT 10-plex (5 μL) was added to the samples and reacted for 1 h at 24 °C. The reactions were quenched with 2 μL of a 5% hydroxylamine solution and combined. The combined mixture was concentrated and fractionated into six samples using a high-pH reversed-phase peptide fractionation kit (Thermo Fisher Scientific). All samples were stored at −20 °C until analysis.

#### Mass Spectrometry Parameters Used for Glycoproteomics and Data Analysis

Again, using established protocols essentially as described by Darabedian et al.,^10,34^ we used the following methods, which are included here for clarity and detail-specific variations. A Thermo Scientific EASY-nLC 1000 system was coupled to an Orbitrap Fusion Tribrid with a nano-electrospray ion source. Mobile phases A and B were water with 0.1% (vol/vol) formic acid and acetonitrile with 0.1% (vol/vol) formic acid, respectively. For the trypsin fractions, peptides were separated using a linear gradient from 4 to 32% B within 50 min, followed by an increase to 50% B within 10 min and further to 98% B within 10 min and re-equilibration. The following instrument parameters were used as previously described.^35^ For the cleavage fractions, peptides were separated with a linear gradient from 5 to 30% B within 95 min, followed by an increase to 50% B within 15 min and further to 98% B within 10 min and re-equilibration. The instrument parameters were set as previously described^34^ with minor modifications. Briefly, MS1 spectra were recorded from *m*/*z* 400–2000 Da. If glyco-fingerprint ions (126.055, 138.055, 144.07, 168.065, 186.076, 204.086, 274.092, and 292.103) were observed in the HCD spectra, ETD (250 ms) with supplemental activation (35%) was performed in a subsequent scan on the same precursor ion selected for HCD. Other relevant parameters of EThcD include: isolation window (3 *m*/*z*), use calibrated charge-dependent ETD parameters (True), orbitrap resolution (50k), first mass (100 *m*/*z*), and inject ions for all available parallelizable time (True). The raw data were processed using Proteome Discoverer 2.4 (Thermo Fisher Scientific). For the trypsin fraction, the data were searched against the UniProt/SwissProt human (*Homo sapiens*) protein database (20,355 proteins, downloaded on Feb 21, 2019) and contaminant proteins using the Sequest HT algorithm. Searches were performed as previously described.^35^ For the cleavage fraction, both HCD and EThcD spectra were searched against the proteome identified in the trypsin fraction using Byonic algorithms. The searches were performed with the following guidelines: trypsin as an enzyme; three missed cleavages allowed; 10 ppm mass error tolerance on precursor ions; and 0.02 Da mass error tolerance on fragment ions. Intact glycopeptide searches allowed for the six most common tagged O-glycan (rare 1) on cysteine, serine, and threonine. Methionine oxidation (common 1) and cysteine carbaminomethylation (common 1) were set as variable modifications with a total common max of 3 and a rare max of 1. Glycopeptide spectral assignments passing an FDR of 1% at the peptide spectrum match level based on a target decoy database were kept. Singly modified glycopeptides assigned from EThCD spectra passing a 1% FDR and possessing a delta modification score of greater than or equal to ten were considered unambiguous glycosites.

### Data Availability

The MS data were deposited at the ProteomeXchange Consortium^36^ via the PRIDE partner repository and are available with the identifier PXD027333.

### hGalK2 Assay

Recombinant human GalK2 was prepared and purified according to the literature.^37^ Recombinant GalK2 (0.005 mg mL^–1^ for GalNAc and 0.05 mg mL^–1^ for 4FGalNAz) was incubated with GalNAc (0.005–0.8 mM) or 4FGalNAz (0.4–20 mM) in triplicate in 1 mL of reaction buffer (60 mM sodium/potassium phosphate, 1.5 mM PEP, 80 mM KCl, 2 mM EDTA, and 10 mM MgCl2, pH 7.0) containing ATP (0.7 mM), NADPH (0.125 mM), pyruvate kinase (35 units), and lactate dehydrogenase (50 units) at 37 °C. Reactions were monitored at 340 nm using a Beckman Coulter DU-640 spectrophotometer.

### AGX1 Protein Expression and Purification

The coding sequence of human AGX1 was cloned into pTriEX 6 with an N-terminus GST-tag (https://doi.org/10.1073/pnas.2007297117), a 3C cleavage site, and a C-terminal FLAG tag, using a BamHI/BglII cloning strategy. A previously established AGX1-FLAG construct was used as a template (https://doi.org/10.1016/j.molcel.2020.03.030), and the primers were CCCTAAGCTTGGATCCCATGAACATTAATGACCTCAAACTCACG (fwd) and GCTCGGTACCAGATCTTCACTTGTCGTCATCGTCTTTGTAGTCAA (rev) for PCR. Plasmid assembly was performed using the In-Fusion HD Cloning Kit (Takara, Kusatsu, Japan). Recombinant baculovirus was generated based on the flashBACTM system (Oxford Expression Technologies, Oxford, UK). Sf21 cells were transfected with a transfer plasmid and flashBAC DNA using Fugene HD (Promega, Madison, USA) according to manufacturer’s instructions.

AGX1 was expressed first by seeding Sf21 cells (2 × 10^6^ cells/mL) and incubating at 27 °C. The following day, cells were infected with viral stocks (P3) using an MOI of 2. After incubation for 3 days, cells were harvested (2000*g*, 5 min, 4 °C) and stored at −80 °C. Pellets were thawed at room temperature, resuspended in 50 mL of cold AGX1 lysis buffer (50 mM Hepes (pH 7.5), 150 mM NaCl, 1 mM EDTA, and 1 mM DTT) with cOmplete protease inhibitors (Roche, Penzberg, Germany) and BaseMuncher mix (1:10,000, Expedeon, Cambridge, UK), and left at 4 °C for 1 h. Cells were then lysed by sonication using a Sonifier 450 (Branson, Hampton, USA) prior to ultracentrifugation (30 000 rpm, 30 min). The supernatant was collected and incubated overnight with 0.5 mL per sample of pre-equilibrated lysis buffer (50 mM Hepes pH 7.5; 150 mM NaCl; 1 mM EDTA; 1 mM DTT, and 10% (v/v) glycerol) and GST-4B Sepharose beads (Sigma-Aldrich, St. Louis, USA). The supernatant was then collected (FT) (2000*g*, 3 min, 4 °C) and washed twice with 10 CV of the same buffer. An aliquot of 100 μL of HRV 3C protease (produced in-house) and 2 CV of lysis buffer containing 10% (v/v) glycerol were added to the beads before incubating at 4 °C for 5 h. The supernatant was collected (E1), and the digestion was repeated three times to obtain E2–E4. E1–E4 were pooled and concentrated to 2 mL using an AmiconTM Ultra15 30K centrifugal tube. The concentrated sample was injected onto an ÄKTATM pure system, running a SuperdexTM S200 16/600 gel filtration column (GE Life Sciences, Marlborough, USA), collecting 1 mL fractions in AGX1 lysis buffer containing 10% (v/v) glycerol. Fractions were pooled and concentrated using an AmiconTM Ultra15 30K centrifugal tube, the concentration was measured using Nanodrop (1.94 mg/mL), and the sample was diluted twice in a freezing buffer (25 mM Hepes pH 7.5; 40% (v/v) glycerol; and 1 mM DTT) and stored at −80 °C.

### AGX1 Assay

Enzyme and time dependence experiments were run to assure initial rates (approx. 5–15% turnover) of reactions. For acceptor substrate GalNAc-1-phosphate Michaelis–Menten kinetics, reaction mixtures containing 5 mM UTP, 0–5 mM GalNAc-1-P, and 4 nM AGX1 and PmPpA (1.6 μg mL^–1^ or 3 U mL^–1^, Chemily Glycoscience) were prepared in buffer containing MgCl2 (5 mM), Tris/HCl (75 mM, pH 8), and BSA (1 mg mL^–1^) in a final volume of 15 μL. Reaction mixtures were incubated at 37 °C for 30–60 min and reactions were stopped by boiling at 95 °C for 10 s and twofold dilution with water. Samples were briefly centrifuged, and supernatants were transferred to a new tube. Samples were run on a UPLC (ACQUITY, Waters) equipped with a UPLC BEH Glycan column (1.7 μm, 2.1 × 100 mm) and a gradient of 90–55% buffer B over 17 min (buffer A: 10 mM ammonium formate, pH 4.5; buffer B: acetonitrile/water 90:10 and 10 mM ammonium formate). Product formation was monitored at 262 nm, confirmed by mass detection in the negative mode and determined by UV peak integration. Data points were calibrated to a standard curve of 0–1.25 mM UDP-GalNAc (Sigma) produced by serial dilution in a final assay buffer. Blanks with an enzymatic mixture and no substrate were included in each set of experiments to account for a potential noise signal at product retention time. Michaelis–Menten parameters were calculated from plots of the initial rate constant at each substrate concentration by nonlinear regression using SigmaPlot 14.0 (Systat Software) of three independent experiments.

Acceptor substrate 4F-GalNAz-1-phosphate kinetics experiments were carried out as mentioned above, except that the reaction mixtures contained 0–10 mM 4F-GalNAz-1-phosphate and 125 nM AGX1. Reaction mixtures were incubated for 2 h and reactions were stopped by the addition of an equal volume of acetonitrile (15 μL) and supernatants were run on UPLC (ACQUITY, Waters) on a gradient of 90–65% buffer B over 17 min. Michaelis–Menten curves were calculated using Prism 9.1 (Graphpad, San Diego, USA) based on three independent experiments.

### GALNT Plasmids

The plasmid hT1-pKN55 encoding a truncated version of human GALNT1 (41–559 amino acids) between the Mlu 1 and Age 1 sites was provided by Lawrence A. Tabak (National Institute of Health). The sequence encoding a truncated version of human GALNT2 (75–572 amino acids) between the Mlu 1 and Age 1 sites from the vector hT2-pIMKF4, provided by Lawrence A. Tabak, was cloned into the Mlu 1-Age 1 sites of the pKN55 vector to create the hT2-pKN55 vector.^38^

### GALNT Expression Screening

The protein was expressed and purified using *Pichia pastoris* according to previously published methods.^39^ Briefly, electroporation-competent *P. pastoris* strain protease-deficient SMD1163 was prepared for electroporation. Vectors were linearized with Sac I and electroporated into competent cells using a Bio-Rad Gene Pulsar set at 1500 V, 25 mF, and 200 Ω. Cells were grown for 3 days at 30 °C on minimal dextrose plates (1.34% yeast nitrogen base, 2% dextrose, and 0.00004% biotin) lacking histidine. Individual colonies were grown in 2 mL of YPG-case medium (1% yeast extract/2% peptone/1.34% yeast nitrogen base/1% glycerol/1% casamino acids/0.00004% biotin/100 mM potassium phosphate, pH 6) in 24-well plates. Cells were grown at 250 rpm in an orbital shaker at 28 °C for 18–24 h and centrifuged at 2000*g* for 5–10 min. The supernatant was replaced with 0.4 mL of YPM-case medium (1% yeast extract/2% peptone/1.34% yeast nitrogen base/0.5% methanol/1%casamino acids/0.00004%biotin/100 mM potassium phosphate, pH 7) to induce protein expression. Cells were cultured for an additional 20–24 h at 20 °C, centrifuged, and the supernatants were analyzed for protein expression by SDS–PAGE. Clones with the best protein expression and enzyme activity were identified and stored as a glycerol stock at −80 °C and streaked onto a fresh MDH (1.34% yeast nitrogen base, 2% dextrose, 0.00004% biotin, and 0.004% histidine) plate as needed. To prepare a culture for a glycerol stock, a loopful of the clone was used to inoculate YPD (1% yeast extract, 2% peptone, and 2% dextrose). The culture was incubated for 24 h at 28 C with shaking. Clones were stored in cryovials with a final glycerol concentration of 25%.

### GALNT Purification

The glycerol stock was streaked onto an MDH agar plate and incubated for 2 days at 28 °C. This plate could be stored at 4 °C for 1 month and used for starter cultures. A loopful of freshly streaked cells from the MDH plate was used to inoculate 5 mL of YPG-case medium, and cells were grown at 250 rpm in an orbital shaker at 28 °C overnight. Next day, the entire starter culture was added to 100 mL of YPG-case medium and cells were grown at 250 rpm in an orbital shaker at 28 °C for 24 h. This 100 mL culture was added to 1 L of YPG-case medium and cells were grown at 250 rpm in an orbital shaker at 28 °C for 24 h until reaching saturation (OD_600_ of ∼20), and then, cells were pelleted in sterile 1 L bottles at 4000 rcf for 30 min. The supernatant was removed, and cell pellets were gently resuspend in 750 mL of YPM-case media without methanol. Cells were cultured at 28 °C for 6 additional hours with shaking to metabolize any remaining glycerol and then induced with 0.5% methanol. Cells were cultured at 20 °C for another 18 h with shaking. Cells were centrifuged at 4000 rcf for 15 min, and the supernatant was collected. Protease inhibitor cocktail (4× sigma fast tablets) was added. The supernatant was concentrated using Amicon filters (MW cutoff 30 KDa), or the protein was salted out using ammonium sulfate. Concentrated protein was resuspended in 50–80 mL of wash buffer 1 (20 mM sodium phosphate pH 7.5; 0.2 M NaCl) and incubated with 20 mL of Ni-NTA beads for 1 h at 4 °C. The supernatant was removed, and the Ni-NTA was washed with wash buffer 1 (2×), followed by wash buffer 2 (20 mM sodium phosphate pH 7.5, 0.2 M NaCl, and 5 mM imidazole). Then, the purified enzyme was eluted using elute buffer (20 mM sodium phosphate pH 7.5; 0.2 M NaCl, and 100 mM imidazole). The eluted enzyme was then desalted and concentrated using Amicon filters (MW cutoff 30KDa) by replacing the elution buffer with 50 mM Tris HCl (pH 7.4) containing 10% glycerol. The GALNT enzyme was aliquoted, flash-frozen, and stored at −80 °C.

### *In Vitro* GALNT Assay

GALNT1 and GALNT2 activity was determined *in vitro* with the UDP-Glo assay (Promega, V6961). The UDP-Glo assay was performed largely as outlined by the manufacturer. Assays were performed in white 96-well plates (Costar, 3912), and reaction volumes were 25 μL. MUC5Ac (GenScript, sequence: GTTPSPVPTTSTTSAP) was used as the peptide acceptor for all reactions. Reaction mixtures contained the following components: 10 nM GALNT1 or 150 nM GALNT2, 50 μM MUC5Ac, 50 μM UDP sugar [ultrapure UDP-GalNAc (Promega, V7081) or UDP-4FGalNAz], and buffer (25 mM Tris–HCl pH 7.4, 10 mM MnCl_2_, 5 mM -mercaptoethanol, and 0.01% Triton). Reaction mixtures were incubated at room temperature for 1 h and then quenched by the addition of 25 μL of UDP-Glo nucleotide detection reagent. The quenched reaction mixtures were mixed briefly by pipetting and incubated in the dark for 1 h at room temperature prior to reading luminescence using BioTek Cytation5. UDP release was quantified using a standard curve of UDP (Promega, V698A). All reactions were run in triplicate. Data were analyzed using Microsoft Excel and Prism 9 (GraphPad).

### *In Vitro* ncOGT Activity Assay

ncOGT activity was determined *in vitro* with the UDP-Glo assay (Promega, V6961) using recombinant ncOGT. ncOGT was purified as described previously.^40^ Briefly, the pET24b plasmid-encoding OGT, provided by Suzanne Walker (Harvard Medical School), was used to produce recombinant OGT in *Escherichia coli*.^41^ ncOGT was purified using an immobilized metal ion affinity chromatography column (Qiagen, 30410) according to the manufacturer’s instructions. Protein purity was estimated by Coomassie staining. ncOGT was >80% pure. The UDP-Glo assay was performed largely as outlined by the manufacturer. Assays were performed in white 96-well plates (Costar, 3912), and reaction volumes were 25 μL. CKII3K (GenScript, sequence: KKKYPGGSTPVSSANMM) was used as the peptide acceptor for all reactions. Reaction mixtures contained the following components: 300 nM ncOGT, 125 μM CKII3K, 40 μM UDP sugar [ultrapure UDP-GlcNAc (Promega, V7071), ultrapure UDP-GalNAc (Promega, V7081), UDP-GlcNAz, or UDP-4FGalNAz], and buffer (150 mM NaCl, 1 mM EDTA, 2.5 mM TCEP, and 25 mM Tris–HCl, pH 7.4). Reaction mixtures were incubated at room temperature for 1 h and quenched by the addition of 25 μL of UDP-Glo nucleotide detection reagent. The quenched reaction mixtures were mixed briefly by pipetting and incubated in the dark for 1 h at room temperature prior to reading luminescence. UDP release was quantified using a standard curve of UDP (Promega, V698A). All reactions were run in triplicate. Data were analyzed using Microsoft Excel and Prism 9 (GraphPad).

### 5SGlcNAc Competition

CHO cells were treated at 20–25% confluency with OGT inhibitor, Ac_4_5SGlcNAc at 200 μM for 24 h before a media change, and treatment with 50 μM Ac_3_4FGalNAz for another 24 h. Cells were collected via scraping before pelleting and lysing as described previously using 4% SDS lysis supplemented with cOmplete, mini, EDTA-free protease inhibitor cocktail tablets (Roche, 5 mg mL^–1^). The lysate was centrifuged for 10 min, 10,000*g* at 4 °C, and protein concentration was determined via BCA assay (Pierce, Thermo Scientific). To 200 μg of protein, freshly made 12 μL of click chemistry cocktail mixture was added after being normalized to 1% SDS and allowed to react in the dark for 1 h before protein precipitation using ice-cold methanol and placed at −20 °C for at least 2 h. The reaction mixtures were spun down (10 min, 10,000*g* at 4 °C), and the supernatant was poured off. The protein pellets were allowed to air-dry for 5–10 min before the addition of 50 μL of 4% SDS buffer and bath sonication to ensure complete dissolution. To the samples, 50 μL of SDS-free 2× loading buffer was added and samples were boiled for 5 min at 95 °C. Proteins were visualized by in-gel fluorescence after 40 μg of protein was loaded per lane for SDS–PAGE separation. After separation, fluorescence was visualized via scanning on a Typhoon 9400 variable mode imager (GE Healthcare) using 532 nm for excitation and a 30 nm band-pass filter centered at 610 nm for detection.

## Supporting Information Available

Supplemental figures, synthetic procedures, and characterization of new compounds (PDF)
Proteomics data (XLSX)

## References

[ref1] Jackson E. G.; Pedowitz N. J.; Pratt M. R. Metabolic Engineering of Glycans. In Comprehensive Glycoscience, 2nd ed.; Barchi J. J., Ed.; Elsevier, 2020; pp 1–13.

[ref2] Pedowitz N. J.; Pratt M. R. Design and Synthesis of Metabolic Chemical Reporters for the Visualization and Identification of Glycoproteins. RSC Chem. Biol. 2021, 2, 306–321. 10.1039/d1cb00010a. 34337414 PMC8323544

[ref3] Nguyen S. S.; Prescher J. A. Developing Bioorthogonal Probes to Span a Spectrum of Reactivities. Nat. Rev. Chem. 2020, 4, 476–489. 10.1038/s41570-020-0205-0. 34291176 PMC8291219

[ref4] Parker C. G.; Pratt M. R. Click Chemistry in Proteomic Investigations. Cell 2020, 180, 605–632. 10.1016/j.cell.2020.01.025. 32059777 PMC7087397

[ref5] Vocadlo D. J.; Hang H. C.; Kim E.-J.; Hanover J. A.; Bertozzi C. R. A Chemical Approach for Identifying O-GlcNAc-Modified Proteins in Cells. Proc. Natl. Acad. Sci. U.S.A. 2003, 100, 9116–9121. 10.1073/pnas.1632821100. 12874386 PMC171382

[ref6] Hang H. C.; Yu C.; Kato D. L.; Bertozzi C. R. A Metabolic Labeling Approach toward Proteomic Analysis of Mucin-Type O-Linked Glycosylation. Proc. Natl. Acad. Sci. U.S.A. 2003, 100, 14846–14851. 10.1073/pnas.2335201100. 14657396 PMC299823

[ref7] Boyce M.; Carrico I. S.; Ganguli A. S.; Yu S.-H.; Hangauer M. J.; Hubbard S. C.; Kohler J. J.; Bertozzi C. R. Metabolic Cross-Talk Allows Labeling of O-Linked β-N-Acetylglucosamine-Modified Proteins via the N-Acetylgalactosamine Salvage Pathway. Proc. Natl. Acad. Sci. U.S.A. 2011, 108, 3141–3146. 10.1073/pnas.1010045108. 21300897 PMC3044403

[ref8] Chuh K. N.; Zaro B. W.; Piller F.; Piller V.; Pratt M. R. Changes in Metabolic Chemical Reporter Structure Yield a Selective Probe of O-GlcNAc Modification. J. Am. Chem. Soc. 2014, 136, 12283–12295. 10.1021/ja504063c. 25153642 PMC4156869

[ref9] Chuh K. N.; Batt A. R.; Zaro B. W.; Darabedian N.; Marotta N. P.; Brennan C. K.; Amirhekmat A.; Pratt M. R. The New Chemical Reporter 6-Alkynyl-6-Deoxy-GlcNAc Reveals O-GlcNAc Modification of the Apoptotic Caspases That Can Block the Cleavage/Activation of Caspase-8. J. Am. Chem. Soc. 2017, 139, 7872–7885. 10.1021/jacs.7b02213. 28528544 PMC6225779

[ref10] Darabedian N.; Gao J.; Chuh K. N.; Woo C. M.; Pratt M. R. The Metabolic Chemical Reporter 6-Azido-6-Deoxy-Glucose Further Reveals the Substrate Promiscuity of O -GlcNAc Transferase and Catalyzes the Discovery of Intracellular Protein Modification by O -Glucose. J. Am. Chem. Soc. 2018, 140, 7092–7100. 10.1021/jacs.7b13488. 29771506 PMC6540071

[ref11] Zaro B. W.; Batt A. R.; Chuh K. N.; Navarro M. X.; Pratt M. R. The Small Molecule 2-Azido-2-Deoxy-Glucose Is a Metabolic Chemical Reporter of O-GlcNAc Modifications in Mammalian Cells, Revealing an Unexpected Promiscuity of O-GlcNAc Transferase. ACS Chem. Biol. 2017, 12, 787–794. 10.1021/acschembio.6b00877. 28135057 PMC6223259

[ref12] Shen D. L.; Liu T.-W.; Zandberg W.; Clark T.; Eskandari R.; Alteen M. G.; Tan H. Y.; Zhu Y.; Cecioni S.; Vocadlo D. Catalytic Promiscuity of O-GlcNAc Transferase Enables Unexpected Metabolic Engineering of Cytoplasmic Proteins with 2-Azido-2-Deoxy-Glucose. ACS Chem. Biol. 2017, 12, 206–213. 10.1021/acschembio.6b00876. 27935279

[ref13] Li J.; Wang J.; Wen L.; Zhu H.; Li S.; Huang K.; Jiang K.; Li X.; Ma C.; Qu J.; et al. An OGA-Resistant Probe Allows Specific Visualization and Accurate Identification of O -GlcNAc-Modified Proteins in Cells. ACS Chem. Biol. 2016, 11, 3002–3006. 10.1021/acschembio.6b00678. 27622469

[ref14] Zaro B. W.; Yang Y.-Y.; Hang H. C.; Pratt M. R. Chemical Reporters for Fluorescent Detection and Identification of O-GlcNAc-Modified Proteins Reveal Glycosylation of the Ubiquitin Ligase NEDD4-1. Proc. Natl. Acad. Sci. U.S.A. 2011, 108, 8146–8151. 10.1073/pnas.1102458108. 21540332 PMC3100932

[ref15] Debets M. F.; Tastan O. Y.; Wisnovsky S. P.; Malaker S. A.; Angelis N.; Moeckl L. K. R.; Choi J.; Flynn H.; Wagner L. J. S.; Bineva-Todd G.; et al. Metabolic Precision Labeling Enables Selective Probing of O-Linked N-Acetylgalactosamine Glycosylation. Proc. Natl. Acad. Sci. U.S.A. 2020, 117, 25293–25301. 10.1073/pnas.2007297117. 32989128 PMC7568240

[ref16] Schumann B.; Malaker S. A.; Wisnovsky S. P.; Debets M. F.; Agbay A. J.; Fernandez D.; Wagner L. J. S.; Lin L.; Li Z.; Choi J.; et al. Bump-and-Hole Engineering Identifies Specific Substrates of Glycosyltransferases in Living Cells. Mol. Cell 2020, 78, 824–834. 10.1016/j.molcel.2020.03.030. 32325029 PMC7276986

[ref17] Yu S.-H.; Boyce M.; Wands A. M.; Bond M. R.; Bertozzi C. R.; Kohler J. J. Metabolic Labeling Enables Selective Photocrosslinking of O-GlcNAc-Modified Proteins to Their Binding Partners. Proc. Natl. Acad. Sci. U.S.A. 2012, 109, 4834–4839. 10.1073/pnas.1114356109. 22411826 PMC3323966

[ref18] Hevey R. Bioisosteres of Carbohydrate Functional Groups in Glycomimetic Design. Biomimetics 2019, 4, 53 10.3390/biomimetics4030053. PMC6784292 31357673

[ref19] Berkin A.; Szarek W. A.; Kisilevsky R. Synthesis of 4-Deoxy-4-Fluoro Analogues of 2-Acetamido-2-Deoxy-d-Glucose and 2-Acetamido-2-Deoxy-d-Galactose and Their Effects on Cellular Glycosaminoglycan Biosynthesis. Carbohydr. Res. 2000, 326, 250–263. 10.1016/s0008-6215(00)00049-5. 10890273

[ref20] Barthel S. R.; Antonopoulos A.; Cedeno-Laurent F.; Schaffer L.; Hernandez G.; Patil S. A.; North S. J.; Dell A.; Matta K. L.; Neelamegham S.; et al. Peracetylated 4-Fluoro-Glucosamine Reduces the Content and Repertoire of N- and O-Glycans without Direct Incorporation. J. Biol. Chem. 2011, 286, 21717–21731. 10.1074/jbc.m110.194597. 21493714 PMC3122228

[ref21] van Wijk X. M.; Thijssen V. L.; Lawrence R.; van den Broek S. A.; Dona M.; Naidu N.; Oosterhof A.; van de Westerlo E. M.; Kusters L. J.; Khaled Y.; et al. Interfering with UDP-GlcNAc Metabolism and Heparan Sulfate Expression Using a Sugar Analogue Reduces Angiogenesis. ACS Chem. Biol. 2013, 8, 2331–2338. 10.1021/cb4004332. 23972127 PMC3821560

[ref22] Wijk X. M.; Lawrence R.; Thijssen V. L.; Broek S. A.; Troost R.; Scherpenzeel M.; Naidu N.; Oosterhof A.; Griffioen A. W.; Lefeber D. J.; et al. A Common Sugar-nucleotide-mediated Mechanism of Inhibition of (Glycosamino)Glycan Biosynthesis, as Evidenced by 6F-GalNAc (Ac3). FASEB J. 2015, 29, 2993–3002. 10.1096/fj.14-264226. 25868729 PMC4478805

[ref23] Walter L. A.; Batt A. R.; Darabedian N.; Zaro B. W.; Pratt M. R. Azide- and Alkyne-Bearing Metabolic Chemical Reporters of Glycosylation Show Structure-Dependent Feedback Inhibition of the Hexosamine Biosynthetic Pathway. Chembiochem 2018, 19, 1918–1921. 10.1002/cbic.201800280. 29979493 PMC6261355

[ref24] Lazarus M. B.; Jiang J.; Gloster T. M.; Zandberg W. F.; Whitworth G. E.; Vocadlo D. J.; Walker S. Structural Snapshots of the Reaction Coordinate for O-GlcNAc Transferase. Nat. Chem. Biol. 2012, 8, 966–968. 10.1038/nchembio.1109. 23103939 PMC3508357

[ref25] Ma X.; Liu P.; Yan H.; Sun H.; Liu X.; Zhou F.; Li L.; Chen Y.; Muthana M. M.; Chen X.; et al. Substrate Specificity Provides Insights into the Sugar Donor Recognition Mechanism of O-GlcNAc Transferase (OGT). PLoS One 2013, 8, e63452 10.1371/journal.pone.0063452. 23700425 PMC3660302

[ref26] Li S.; Wang J.; Zang L.; Zhu H.; Guo J.; Zhang J.; Wen L.; Chen Y.; Li Y.; Chen X.; et al. Production of Glycopeptide Derivatives for Exploring Substrate Specificity of Human OGA Toward Sugar Moiety. Front. Chem. 2019, 6, 646 10.3389/fchem.2018.00646. 30693278 PMC6340312

[ref27] Qin W.; Qin K.; Fan X.; Peng L.; Hong W.; Zhu Y.; Lv P.; Du Y.; Huang R.; Han M.; et al. Artificial Cysteine S-Glycosylation Induced by Per-O-Acetylated Unnatural Monosaccharides during Metabolic Glycan Labeling. Angew. Chem., Int. Ed. 2018, 57, 1817–1820. 10.1002/anie.201711710. 29237092

[ref28] Qin K.; Zhang H.; Zhao Z.; Chen X. Protein S-Glyco-Modification through an Elimination–Addition Mechanism. J. Am. Chem. Soc. 2020, 142, 9382–9388. 10.1021/jacs.0c02110. 32339456

[ref29] Woo C. M.; Iavarone A. T.; Spiciarich D. R.; Palaniappan K. K.; Bertozzi C. R. Isotope-Targeted Glycoproteomics (IsoTaG): A Mass-Independent Platform for Intact N- and O-Glycopeptide Discovery and Analysis. Nat. Methods 2015, 12, 561–567. 10.1038/nmeth.3366. 25894945 PMC4599779

[ref30] Woo C. M.; Felix A.; Byrd W. E.; Zuegel D. K.; Ishihara M.; Azadi P.; Iavarone A. T.; Pitteri S. J.; Bertozzi C. R. Development of IsoTaG, a Chemical Glycoproteomics Technique for Profiling Intact N- and O-Glycopeptides from Whole Cell Proteomes. J. Proteome Res. 2017, 16, 1706–1718. 10.1021/acs.jproteome.6b01053. 28244757 PMC5507588

[ref31] Ju T.; Cummings R. D. A Unique Molecular Chaperone Cosmc Required for Activity of the Mammalian Core 1 Beta 3-Galactosyltransferase. 2002, 99 (), 16613–16618. 10.1073/pnas.262438199 PMC139192 12464682

[ref32] Zhai Y.; Liang M.; Fang J.; Wang X.; Guan W.; Liu X.-w.; Wang P.; Wang F. NahK/GlmU Fusion Enzyme: Characterization and One-Step Enzymatic Synthesis of UDP-N-Acetylglucosamine. Biotechnol. Lett. 2012, 34, 1321–1326. 10.1007/s10529-012-0910-y. 22456903

[ref33] Cejas R. B.; Lorenz V.; Garay Y. C.; Irazoqui F. J. Biosynthesis of O-N-Acetylgalactosamine Glycans in the Human Cell Nucleus. J. Biol. Chem. 2019, 294, 2997–3011. 10.1074/jbc.ra118.005524. 30591584 PMC6398145

[ref34] Darabedian N.; Yang B.; Ding R.; Cutolo G.; Zaro B. W.; Woo C. M.; Pratt M. R. O-Acetylated Chemical Reporters of Glycosylation Can Display Metabolism-Dependent Background Labeling of Proteins but Are Generally Reliable Tools for the Identification of Glycoproteins. Front. Chem. 2020, 8, 318 10.3389/fchem.2020.00318. 32411667 PMC7198827

[ref35] Ge Y.; Ramirez D. H.; Yang B.; D’Souza A. K.; Aonbangkhen C.; Wong S.; Woo C. M. Target Protein Deglycosylation in Living Cells by a Nanobody-Fused Split O-GlcNAcase. Nat. Chem. Biol. 2021, 17, 593–600. 10.1038/s41589-021-00757-y. 33686291 PMC8085020

[ref36] Vizcaíno J. A.; Deutsch E. W.; Wang R.; Csordas A.; Reisinger F.; Ríos D.; Dianes J. A.; Sun Z.; Farrah T.; Bandeira N.; Binz P.-A.; Xenarios I.; Eisenacher M.; Mayer G.; Gatto L.; Campos A.; Chalkley R. J.; Kraus H.-J.; Albar J. P.; et al. ProteomeXchange Provides Globally Coordinated Proteomics Data Submission and Dissemination. Nat. Biotechnol. 2014, 32, 223–226. 10.1038/nbt.2839. 24727771 PMC3986813

[ref37] Thoden J. B.; Holden H. M. The Molecular Architecture of Human N-Acetylgalactosamine Kinase. J. Biol. Chem. 2005, 280, 32784–32791. 10.1074/jbc.m505730200. 16006554

[ref38] Fritz T. A.; Hurley J. H.; Trinh L.-B.; Shiloach J.; Tabak L. A. The Beginnings of Mucin Biosynthesis: The Crystal Structure of UDP-GalNAc:Polypeptide {alpha}-N-Acetylgalactosaminyltransferase-T1. Proc. Natl. Acad. Sci. U.S.A. 2004, 101, 15307–15312. 10.1073/pnas.0405657101. 15486088 PMC524453

[ref39] Dikiy I.; Clark L. D.; Gardner K. H.; Rosenbaum D. M. Isotopic Labeling of Eukaryotic Membrane Proteins for NMR Studies of Interactions and Dynamics. Methods Enzymol. 2019, 614, 37–65. 10.1016/bs.mie.2018.08.030. 30611431 PMC7309954

[ref40] Rodriguez A. C.; Kohler J. J. Recognition of Diazirine-Modified O-GlcNAc by Human O-GlcNAcase. Medchemcomm 2014, 5, 1227–1234. 10.1039/c4md00164h. 25068034 PMC4109824

[ref41] Lazarus M. B.; Nam Y.; Jiang J.; Sliz P.; Walker S. Structure of Human O-GlcNAc Transferase and Its Complex with a Peptide Substrate. Nature 2011, 469, 564–567. 10.1038/nature09638. 21240259 PMC3064491

