# Supplementary material for: 4-Deoxy-4-fluoro-GalNAz (4FGalNAz) Is a Metabolic Chemical Reporter of O-GlcNAc Modifications, Highlighting the Notable Substrate Flexibility of O-GlcNAc Transferase
Source: ACS Chem Biol. 2021 Dec 21;17(1):159–70. doi: 10.1021/acschembio.1c00818 (PMC8787749; doi:10.1021/acschembio.1c00818)
Supplement: Supplementary file 1 — cb1c00818_si_001.pdf [file cb1c00818_si_001.pdf]

4-deoxy-4-fluoro-GalNAz (4FGalNAz) is a metabolic chemical reporter of O-GlcNAc modifications, highlighting the notable substrate flexibility of O-GlcNAc transferase.

Emma G. Jackson,[1,#] Giuliano Cutolo,[1,#] Bo Yang,[2] Nageswari Yarravarapu,[3] Mary W. N. Burns,[3] Ganka Bineva-Todd,[4] Chloë Roustan,[5] James B. Thoden,[6] Halley M. Lin-Jones,[1] Toin H. van Kuppevelt,[7] Hazel M. Holden,[6] Benjamin Schumann,[4,8] Jennifer J. Kohler,[3] Christina M. Woo,[2] and Matthew R. Pratt[1,9,*]

[1]Departments of Chemistry and [9]Biological Sciences, University of Southern California, Los Angeles, CA 90089, United States
[2]Department of Chemistry and Chemical Biology, Harvard University, Cambridge, Massachusetts, 02138, United States
[3]Department of Biochemistry, University of Texas Southwestern Medical Center, Dallas, TX 75390, United States
[4]Chemical Glycobiology Laboratory, The Francis Crick Institute, NW1 1AT London, United Kingdom.
[5]Structural Biology Science Technology Platform, The Francis Crick Institute, NW1 1AT London, United Kingdom
[6]Department of Biochemistry, University of Wisconsin, Madison, WI 53706, United States
[7]Department of Biochemistry, Radboud Institute for Molecular Life Sciences, Radboud University Medical Centre, Nijmegen, 6500 HB, The Netherlands.
[8]Department of Chemistry, Imperial College London, W120BZ London, United Kingdom

*Corresponding author: Matthew R. Pratt, matthew.pratt@usc.edu
[#]These authors contributed equally

Table of contents:

Figure S1. Synthesis of $Ac_3$4FGalNAz **Page S2**
Figure S2. 4FGalNAz is not particularly toxic to mammalian cells. **Page S2**
Figure S3. Characterization of 4FGalNAz in Jurkat cells. **Page S3**
Figure S4. 4FGalNAz is a substrate for GalK2 and AGX1. **Page S3**

Experimental Methods **Page S4**

NMR characterization of synthetic compounds **Page S7**

References **Page S11**

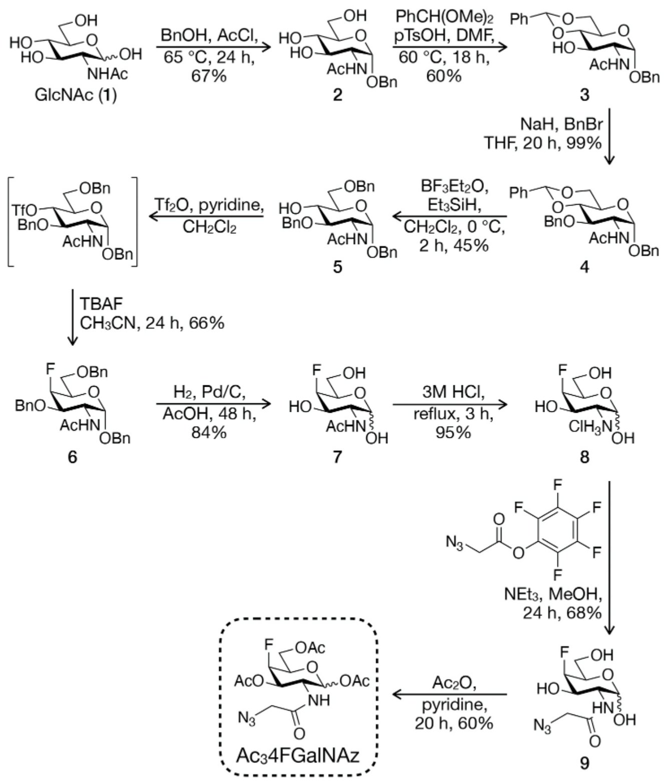

Figure S1. Synthesis of $Ac_3$4FGalNAz.

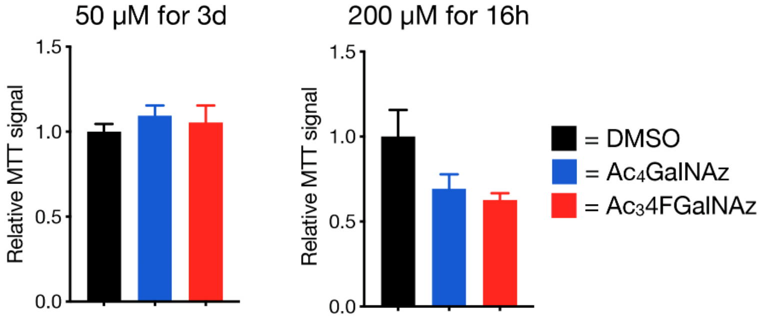

Figure S2. 4FGalNAz is not particularly toxic to mammalian cells. CHO cells were incubated with MCR or DMSO vehicle under the indicated conditions before cell viability was measured using an MTT assay.

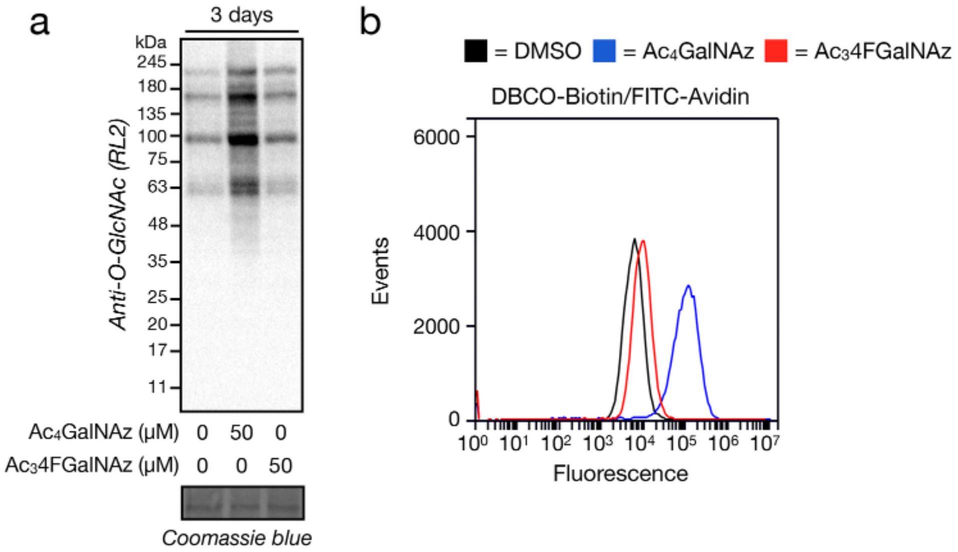

Figure S3. Characterization of 4FGalNAz in Jurkat cells. a) 4FGalNAz does not inhibit O-GlcNAc modifications. Jurkat cells were treated under the indicated conditions before visualization of O-GlcNAc levels by western blotting. b) 4FGalNAz labeling can be detected by flow cytometry. Jurkat cells were treated with individual MCRs (50 µM) for 3 d before the live cells were subjected to SPACC with DBCO-biotin, followed by FITC-Avidin and detection of fluorescence by flow-cytometry.

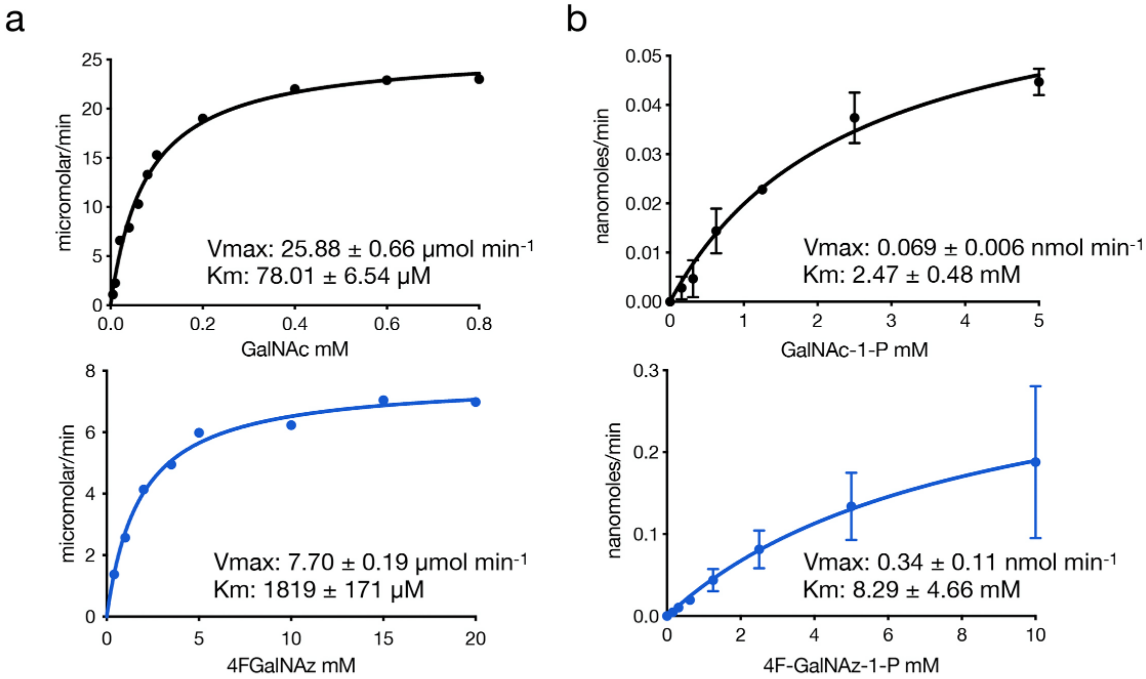

Figure S4. 4FGalNAz is a substrate for GalK2 and AGX1. a) 4FGalNAz is accepted by GALK2. Michaelis-Menten enzyme curves were measured using recombinant GalK2 and the indicated concentrations of either GalNAc or 4FGalNAz. Enzyme constants were determined using line fitting in Graphpad Prism 9. b) 4FGalNAz-1-phosphate is accepted by AGX1. Michaelis-Menten enzyme curves were measured using recombinant AGX1 and the indicated concentrations of either GalNAc or 4FGalNAz. Enzyme constants were determined using line fitting in Graphpad Prism 9.

Experimental Methods

Chemical synthesis

General information

All reagents used for chemical synthesis were purchased from Sigma-Aldrich, Alfa Aesar or EMD Millipore unless otherwise specified and used without further purification. All anhydrous reactions were performed under argon or nitrogen atmosphere. Analytical thin-layer chromatography (TLC) was conducted on EMD Silica Gel 60 F254 plates with detection by ceric ammonium molybdate (CAM), anisaldehyde or UV. For flash chromatography, 60 Å silica gel (EMD) was utilized. ^{1}H spectra were obtained at 400, 500, or 600 MHz on Varian spectrometers Mercury 400, VNMRS-500, or -600. Chemical shifts are recorded in ppm (δ) relative to solvent. Coupling constants (*J*) are reported in Hz. ^{13}C spectra were obtained at 100, 125, or 150 MHz on the same instruments.

1-O-Benzyl-N-acetyl-α-D-glucosamine (2)[1]

To a stirred suspension of *N*-acetylglucosamine **1** (10.0 g, 45.2 mmol) in benzyl alcohol (125 mL) at 0 °C, acetyl chloride (10.9 mL, 12.0 g, 152 mmol) was added dropwise. The reaction mixture was stirred for 30 min at rt and further stirred for 24 h at 65 °C. The benzyl glycoside was precipitated using cold Et_2O and the liquid phase was discarded. The resulting syrup corresponding to the benzyl glycoside was washed with cold Et_2O, solubilized in MeOH and neutralized with $NaHCO_3$ (solid) until pH 7 was achieved. The suspension was filtered through a short pad of Celite™, further washed with MeOH, and the solvent was removed under reduced pressure. Recrystallization from EtOH yielded **2** (11.0 g, 78%) as a white solid. ^{1}H NMR (400 MHz, CD_3OD) δ 7.43 – 7.20 (m, 5H), 4.74 (d, *J* = 12.0 Hz, 1H), 4.59 (s, 1H), 4.49 (d, *J* = 12.0 Hz, 1H), 4.00 – 3.56 (m, 4H), 3.41 – 3.27 (m, 2H), 1.94 (s, 3H). HRMS: calc'd. for $C_{15}H_{21}NNaO_6$ $(M+Na)^+$ 334.1267, found 334.1262.

Benzyl 2-Acetamido-4,6-O-benzylidene-2-deoxy-α-D-glucopyranoside (3)[1]

Benzaldehyde (3.46 ml, 23.0 mmol) and *p*-toluenesulfonic acid (2 g, 11.5 mmol) were added to a suspension of the starting material **2** (6.5 g, 20.88 mmol) in anhydrous DMF (20 ml). The mixture was stirred at 65°C for 20 h. After this time the solvent was evaporated under reduced pressure, then the solid residue washed and triturated with hexane. The residue was then triturated with a warm $NaHCO_3$ saturated solution. After cooling to room temperature, the solid was filtered and the solvent evaporated to afford the compound **3** (7.6 g, 91%) as a solid. ^{1}H NMR (400 MHz, $CDCl_3$) δ 7.56 – 7.45 (m, 5H), 7.43 – 7.31 (m, 5H), 5.85 (d, *J* = 8.9 Hz, 1H), 5.56 (s, 1H), 4.93 (d, *J* = 3.8 Hz, 1H), 4.77 – 4.67 (m, 1H), 4.49 (d, *J* = 11.8 Hz, 1H), 4.28 – 4.17 (m, 2H), 3.95 (t, *J* = 9.6 Hz, 1H), 3.90 – 3.83 (m, 1H), 3.76 (t, *J* = 10.2 Hz, 1H), 3.65 – 3.55 (m, 2H), 1.99 (s, 3H). HRMS: calc'd. for $C_{22}H_{25}NNaO_6$ $(M+Na)^+$ 422.1580, found 422.1564.

Benzyl 2-Acetamido-3-O-benzyl-4,6-O-benzylidene-2-deoxy-α-D-glucopyranoside (4)[2]

NaH 60% suspension in mineral oil (1 g, 25.0 mmol) was added portionwise to a solution of the derivative **3** (5 g, 12.5 mmol) and benzyl bromide (3 mL, 25.0 mmol) in THF (150 mL) at 0°C. The reaction mixture was stirred for 24 h, then the reaction was quenched in cold water and neutralized with formic acid 10%. The solid was filtered, washed with water and dried under reduced pressure to yield the compound **4** (4.60 g, 75%). ^{1}H NMR (400 MHz, $CDCl_3$) δ 7.58 – 7.19 (m, 15H), 5.60 (s, 1H), 5.35 (d, *J* = 9.3 Hz, 1H), 4.92 (dd, *J* = 8.1, 4.2 Hz, 2H), 4.67 (dd, *J* = 27.8, 12.0 Hz, 2H), 4.46 (d, *J* = 11.8 Hz, 1H), 4.31 (td, *J* = 9.3, 3.8 Hz, 1H), 4.25 (dd, *J* = 10.1, 4.7 Hz, 1H), 3.94 – 3.85 (m, 1H), 3.83 – 3.71 (m, 2H), 1.87 (s, 3H).

Benzyl 2-Acetamido-3,6-di-O-benzyl-2-deoxy-α-D-glucopyranoside (5)[2]

BF_3/Et_2O (1.55 mL, 12.6 mmol) was added dropwise to a solution of Et_3SiH (6.7 mL, 42.0 mmol) and compound **4** (4.11 g, 8.40 mmol) in dry CH2Cl2 (47 mL) at 0 °C. After stirring at 0 °C for 2 h, the reaction mixture was quenched with triethylamine until neutralization, and then purified through flash silica gel column (toluene:acetone 7:3) to yield compound **5** (2.6 g, 63%) as a white solid. ^{1}H NMR (400 MHz, $CDCl_3$) δ 7.43 – 7.23 (m, 15H), 5.42 (d, *J* = 9.3 Hz, 1H), 4.89 (d, *J* = 3.8, 1.2 Hz, 1H), 4.79 – 4.67 (m, 3H), 4.59 (q, *J* = 11.9 Hz, 2H), 4.45 (d, *J* = 11.7 Hz, 1H), 4.27 (td, *J* = 10.4, 9.9, 3.7 Hz, 1H), 3.84 – 3.59 (m, 4H), 1.85 (d, *J* = 1.2 Hz, 3H).

Benzyl 2 -acetamido-3,6 -di-O-benzyl-2,4-dideoxy-4-fluoro-α-D-galactopyranoside (6)[3]

*To a solution of the compound **5** (4.6 g, 9.36 mmol) in anhydrous DCM (93 mL) and anhydrous pyridine (15.5 mL) stirred at 0 °C was added triflic anhydride (3.26 mL, 19.65 mmol) dropwise. The solution was stirred under the same conditions for 1 h. After that time, the reaction was diluted with DCM, washed with HCl 1M x2, saturated aqueous $NaHCO_3$, and brine solution. The organic phase was dried over Na_2SO_4, and evaporated under reduced pressure. The residue was engaged in the next step without further purification. Tetra-N-butylammonium fluoride (19.6 g, 74.91 mmol) was added to a solution of the crude triflate derivative (5.84 g, 9.36mmol) in anhydrous MeCN (172 mL). The mixture was stirred at room temperature for 24 h. After evaporation of the solvent, the crude reaction mixture was subjected to flash chromatography on silica gel (hexane:acetone 6:4). The resulting impure glycoside was further purified by reversed phase C-18 column chromatography (H_2O:ACN 60:40 to 0:100 0.1% TFA in 24 min) to afford the compound **6** (2.86 g, 62% over 2 steps). ^1H NMR (400 MHz, $CDCl_3$) δ 7.37 – 7.19 (m, 15H), 5.36 (d, J = 9.0 Hz, 1H), 4.97 (d, J = 3.7 Hz, 1H), 4.77 – 4.62 (m, 3H), 4.54 (s, 3H), 4.46 (dd, J = 12.0, 7.5 Hz, 2H), 3.74 – 3.55 (m, 3H), 1.87 (s, 3H). HRMS: calc'd. for $C_{29}H_{33}FNO_5$ $(M+H)^+$ 494.2343, found 494.2324.*

2-Acetamido-2,4-dideoxy-4-fluoro-D-galactopyranose (7)[4]

Pd/C (10%, 2.4 g) was added to a solution of **6** (2.86 g, 5.79 mmol) in acetic acid (83 mL). The mixture was vigorously stirred under H_2 atmosphere for 48 h. After completion by TLC the mixture was filtered over a celite pad and the solvent evaporated in vacuo. The mixture was purified on a RP C-18 column chromatography (H_2O:ACN 100:0 to 0:100 0.1% TFA in 15 min) to yield the compound **7** (1.09 g, 84%). ^1H NMR (400 MHz, CD_3OD) δ 5.12 (d, *J* = 3.5 Hz, 1H), 4.17 (dd, *J* = 11.1, 3.5 Hz, 1H), 4.12 – 3.98 (m, 1H), 3.95 – 3.83 (m, 1H), 3.72 – 3.59 (m, 3H). ^{19}F NMR (376 MHz, CD_3OD) δ -223.29 – -223.68 (m).

2-Amino-2,4-dideoxy-4-fluoro-D-galactose hydrochloride (8)[4]

A stirred solution of **7** (1.25 g, 5.60 mmol) in 3M HCl (56 mL) was heated at 95-100°C for 3h. After this time the solvent was evaporated, and the crude was eluted on a RP C-18 flash chromatography (H_2O:ACN 100:0 to 0:100 0.1% TFA over 15 min) to yield **8** (1.1 g, 90%). ^1H NMR (400 MHz, CD_3OD) δ 5.43 (d, *J* = 3.5 Hz, 1H), 4.19 – 4.01 (m, 2H), 3.74 – 3.63 (m, 3H), 3.42 – 3.34 (m, 1H). ^{19}F NMR (376 MHz, CD_3OD) δ -223.89 – -224.34 (m). HRMS: calc'd. for $C_6H_{13}FNO_4$ $(M-Cl)^+$ 182.0829, found 182.0828.

2-azidoacetamido-2,4-dideoxy-4-fluoro-D-galactopyranose (9)

Pentafluorophenyl Trifluoromethanesulfonate (3.4 mL, 19.79 mmol) was added to a solution of azido acetic acid (1 g, 9.89 mmol) and anhydrous pyridine (3.6 mL, 44.53 mmol) in DMF (20 mL), then the solution was stirred at room temperature for 18h. The solvent was partially evaporated and the residue was diluted with Et_2O and washed subsequentially with 2x aqueous saturated $NaHCO_3$ and brine. The organic phase was dried over Na_2SO_4, and the solvent evaporated under reduced pressure. The crude mixture was then subjected to silica gel flash column chromatography (Hexane:Acetone 8:2) to afford the azido acetate pentafluorophenyl ester. (2 g, 73%) The azido acetate pentafluorophenyl ester (1.96 g, 7.35 mmol) was added to a mixture of the compound **8** (800 mg, 3.68 mmol), and triethylamine (1.3 mL, 9.19 mmol) in MeOH (20 mL). The mixture was stirred at room temperature for 24 h. The solvent was then evaporated under reduced pressure and the crude mixture purified by RP C-18 flash chromatography (H_2O:ACN 100:0 to 0:100 0.1% TFA in 25 min) to afford the compound **9**. (950 mg, 98%). ^1H NMR (400 MHz, CD_3OD) δ 5.13 (d, *J* = 3.5 Hz, 1H), 4.20 (dd, *J* = 11.0, 3.6 Hz, 1H), 4.06 (dt, *J* = 30.3, 6.7 Hz, 1H), 3.94 – 3.83 (m, 4H), 3.72 – 3.62 (m, 2H). ^{19}F NMR (376 MHz, CD_3OD) δ -222.77 (dt, *J* = 50.8, 29.6 Hz). ^{13}C NMR (101 MHz, CD_3OD) δ 169.15, 91.28, 88.15, 71.28, 69.35, 66.68, 61.33, 51.38. HRMS: calc'd. for $C_8H_{13}FN_4NaO_5$ $(M+Na)^+$ 287.0768, found 287.0766.

1, 3, 6-tri-O-acetyl-2-azidoacetamido-2,4-dideoxy-4-fluoro-D-galactopyranose (10)

Acetic anhydride (4 mL) was added to a solution of the compound **9** (950 mg, 3.60 mmol) in pyridine (8 mL); and the solution was stirred at room temperature for 24 h. The residue was co-evaporated with toluene, then purified by silica gel column chromatography (hexane:acetone 7:3) to afford the compound **10** (900 mg, 61%) as a yellow-orange amorphous solid. ^1H NMR (400 MHz, $CDCl_3$) δ 6.41 (d, *J* = 8.9 Hz, 1H), 6.23 (d, *J* = 3.6 Hz,

1H), 5.31 – 5.14 (m, 2H), 4.79 – 4.68 (m, 1H), 4.31 – 4.13 (m, 2H), 3.95 (s, 2H), 2.17 (s, 3H), 2.14 (s, 3H), 2.06 (s, 3H). ^{19}F NMR (376 MHz, $CDCl_3$) δ -213.65 – -214.08 (m).^{13}C NMR (101 MHz, $CDCl_3$) δ 171.35, 170.47, 168.81, 167.20, 90.73, 86.66, 69.07, 67.93, 61.37, 52.40, 47.02, 20.87, 20.78, 20.70. HRMS: calc'd. for $C_{14}H_{20}FN_4O_8$ $(M+H)^+$ 391.1265, found 391.1268.

2-azidoacetamido-4-fluoro-2, 4-dideoxy-D-galactopyranose 1-(dihydrogen phosphate)
A 1750 µL solution of trGlmU– NahK[5] (3.5 mg), 4FGalNAz (**9**) (9.3 mg, 20 mM), ATP (10 mM) and $MgCl_2$ (5 mM), in 200 mM Tris/HCl buffer (pH 8.0) was incubated at 37 °C for 24 h. The pH of the solution was verified using pH indicator paper before the addition of the enzyme. After 24 h, the reaction was lyophilized, then resuspended in 1:1 ACN:H_2O and purified by HPLC SeQuant ZIC-HILIC chromatography column (5 µm, 200A, 150 x 10 mm, EMD Milipore), using a gradient 10% to 40% B over 35 min, (buffer A: ACN, buffer B: 20 mM NH_4OAc in H_2O) to give the 1-phophate product (2.7 mg, 40%). ^{1}H NMR (600 MHz, D_2O δ 5.37 – 5.33 (m, 1H), 4.83 (d, J = 50.8 Hz, 1H), 4.21 – 4.10 (m, 2H), 4.01 (d, J = 16.3 Hz, 1H), 3.94 (d, J = 16.1 Hz, 1H), 3.71 – 3.66 (m, 1H), 1.79 (s, 2H). ^{31}P NMR (243 MHz, D_2O) δ 0.86. ^{19}F NMR (564 MHz, D_2O) δ -220.66 (dt, J = 50.7, 30.5 Hz). ^{13}C NMR (151 MHz, D_2O) δ 181.35, 171.05, 92.99, 90.23, 70.01, 60.06, 51.68, 23.16. HRMS: calc'd. for $C_8H_{13}FN_4O_8P$ $(M-H)^-$ 343.0455, found 343.0459.

Uridine 5′- Diphospho-(2-azidoacetamido-4-fluoro-1, 2, 4-dideoxy-D-galactopyranosyl)
A 2500 µL solution of trGlmU– NahK (5 mg), 4FGalNAz-1-phosphate (17.2 mg, 20 mM), ATP (10 mM),UTP (10 mM), inorganic pyrophosphatase (5 Units) and $MgCl_2$ (5 mM), in 200 mM Tris/HCl buffer (pH 8.0) was incubated at 37 °C for 24 h. The pH of the solution was verified using pH indicator paper before the addition of the enzyme. After 24 h, the reaction was lyophilized, then resuspended in 5:5 ACN:H_2O and purified by HPLC SeQuant ZIC-HILIC chromatography column (5 µm, 200A, 150 x 10 mm, EMD Milipore), using a gradient 10% to 40% B over 35 min, (buffer A: ACN, buffer B: 20 mM NH_4OAc in H_2O) to give UDP-4FGalNaz (3 mg, 28%). ^{1}H NMR (400 MHz, D_2O) δ 7.80 (d, J = 8.1 Hz, 1H), 5.85 – 5.78 (m, 1H), 4.30 – 3.87 (m, 4H), 3.72 – 3.55 (m, 2H), 3.53 – 3.35 (m, 2H), 3.20 (s, 2H), 1.76 (s, 6H), 1.19 – 1.08 (m, 1H). ^{31}P NMR (162 MHz, D_2O) δ -11.40 (d, J = 20.5 Hz), -13.23 (d, J = 21.1 Hz). ^{19}F NMR (376 MHz, D_2O) δ -220.79 (dt, J = 50.0, 30.2 Hz). ^{13}C NMR (151 MHz, D_2O) δ 171.13, 166.21, 151.79, 141.65, 102.60, 94.34, 89.22 (d, J = 210.6 Hz), 83.15, 83.09, 73.69, 72.02, 69.61, 65.00, 62.44, 59.84, 51.57, 50.03. HRMS: calc'd. for $C_{17}H_{24}FN_6O_{16}P_2$ $(M-H)^-$ 649.0708, found 649.0706.

2-azidoacetamido-2,4-dideoxy-4-fluoro-D-galactopyranose (9)

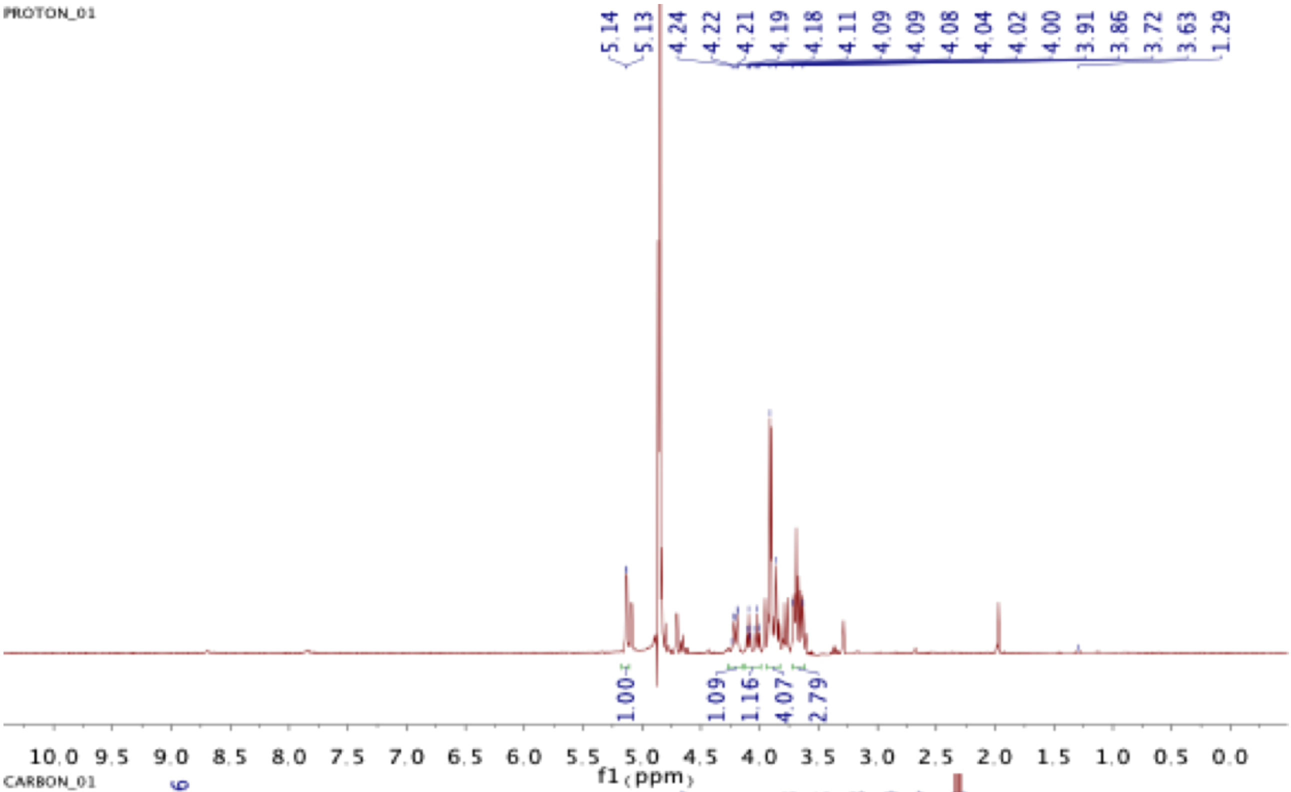

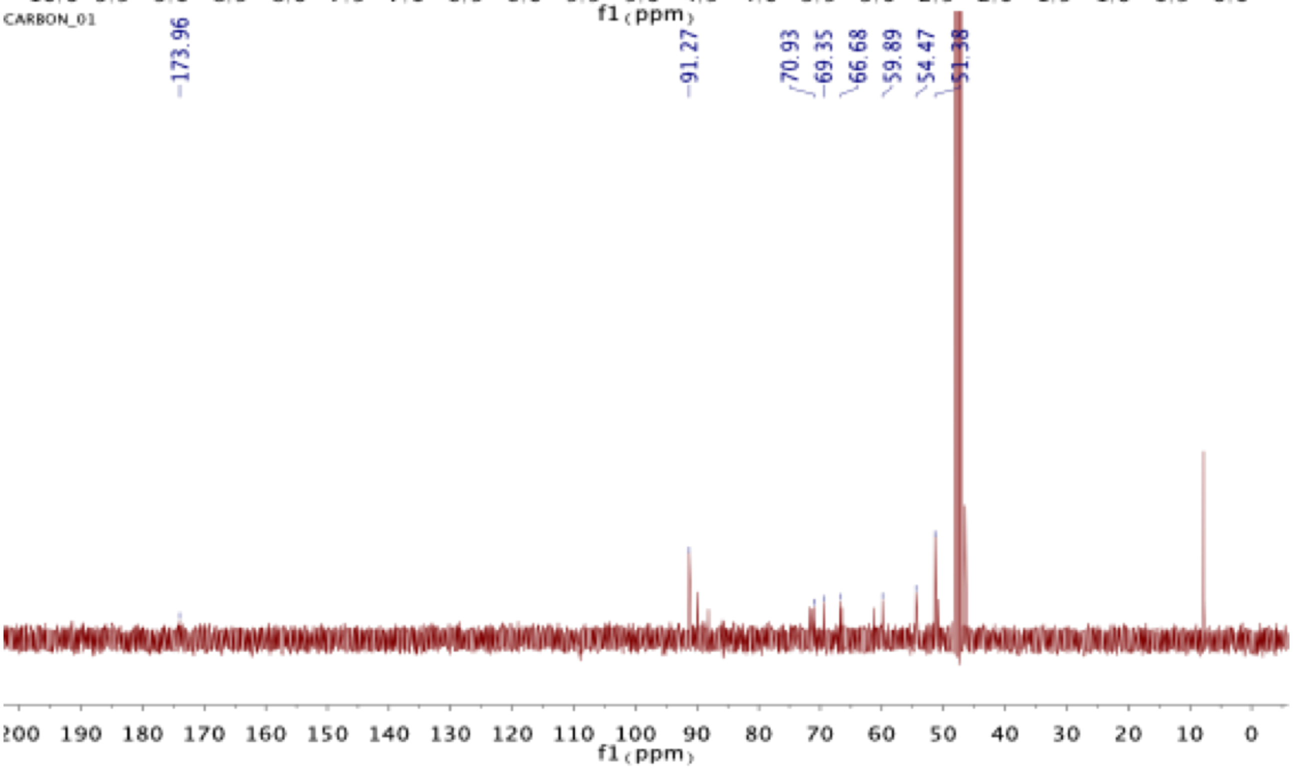

1, 3, 6-tri-O-acetyl-2-azidoacetamido-2,4-dideoxy-4-fluoro-D galactopyranose (Ac$_3$4FGalNAz)

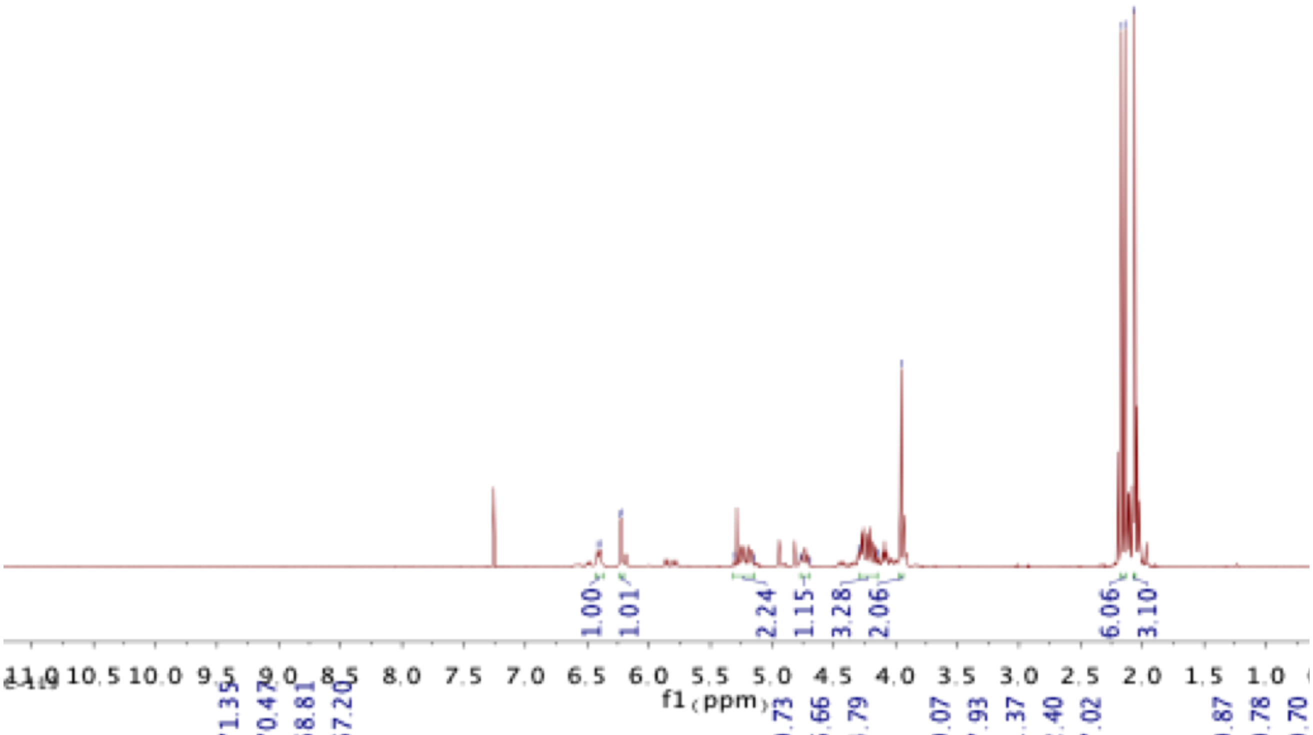

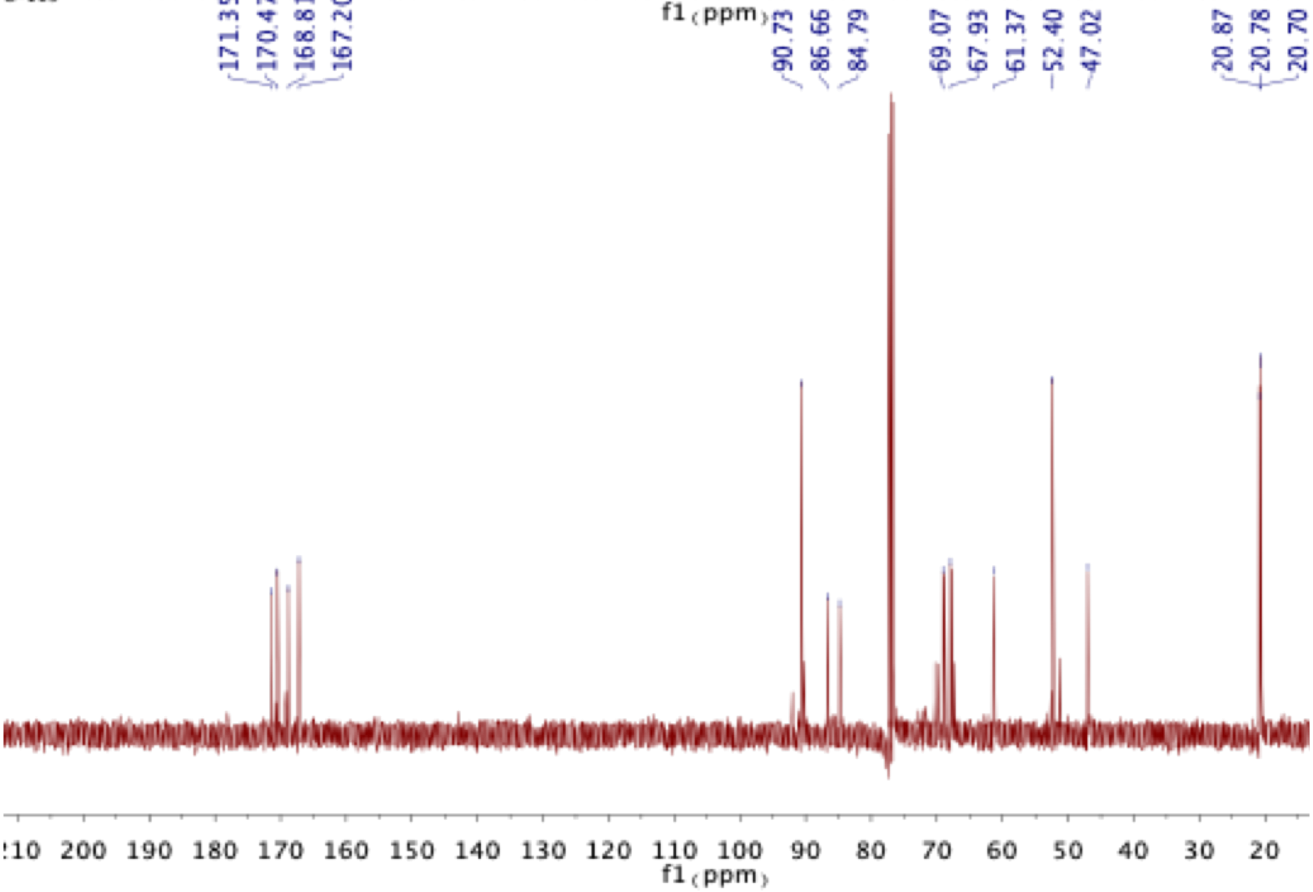

2-azidoacetamido-4-fluoro-2, 4-dideoxy-D-galactopyranose 1-(dihydrogen phosphate) (4FGalNAz-1-phosphate)

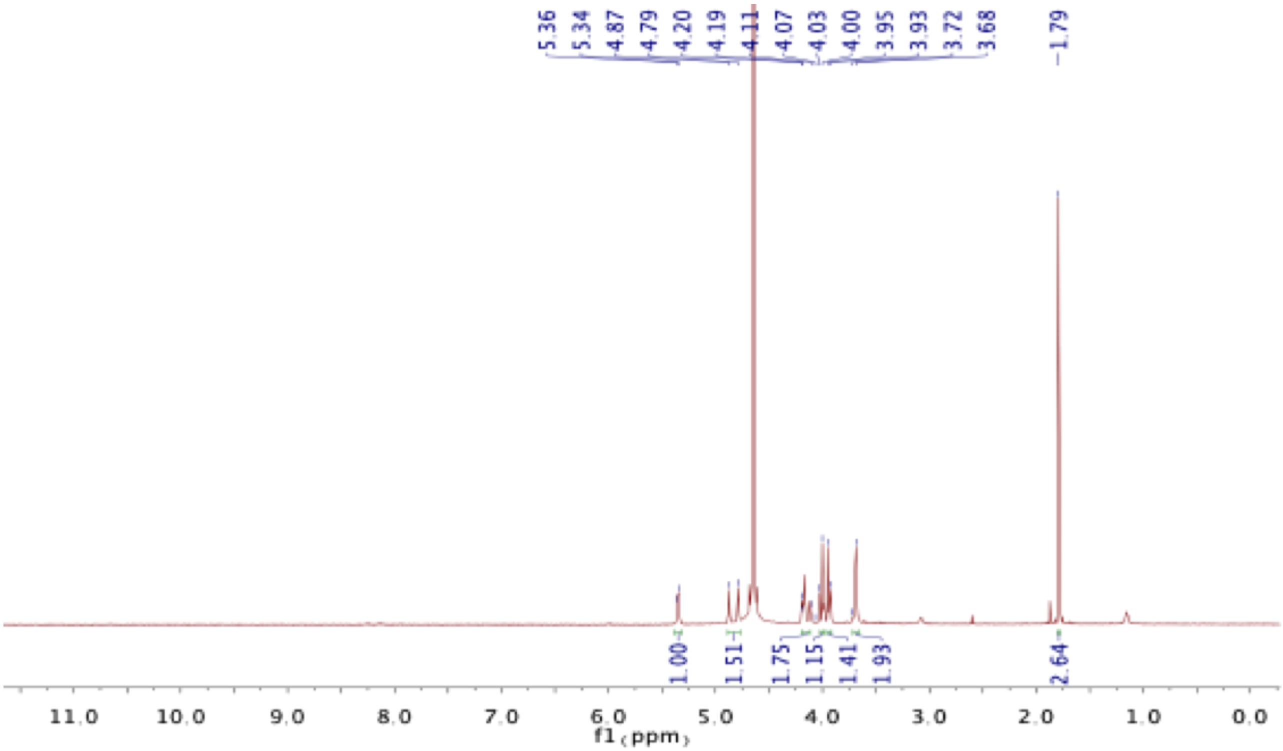

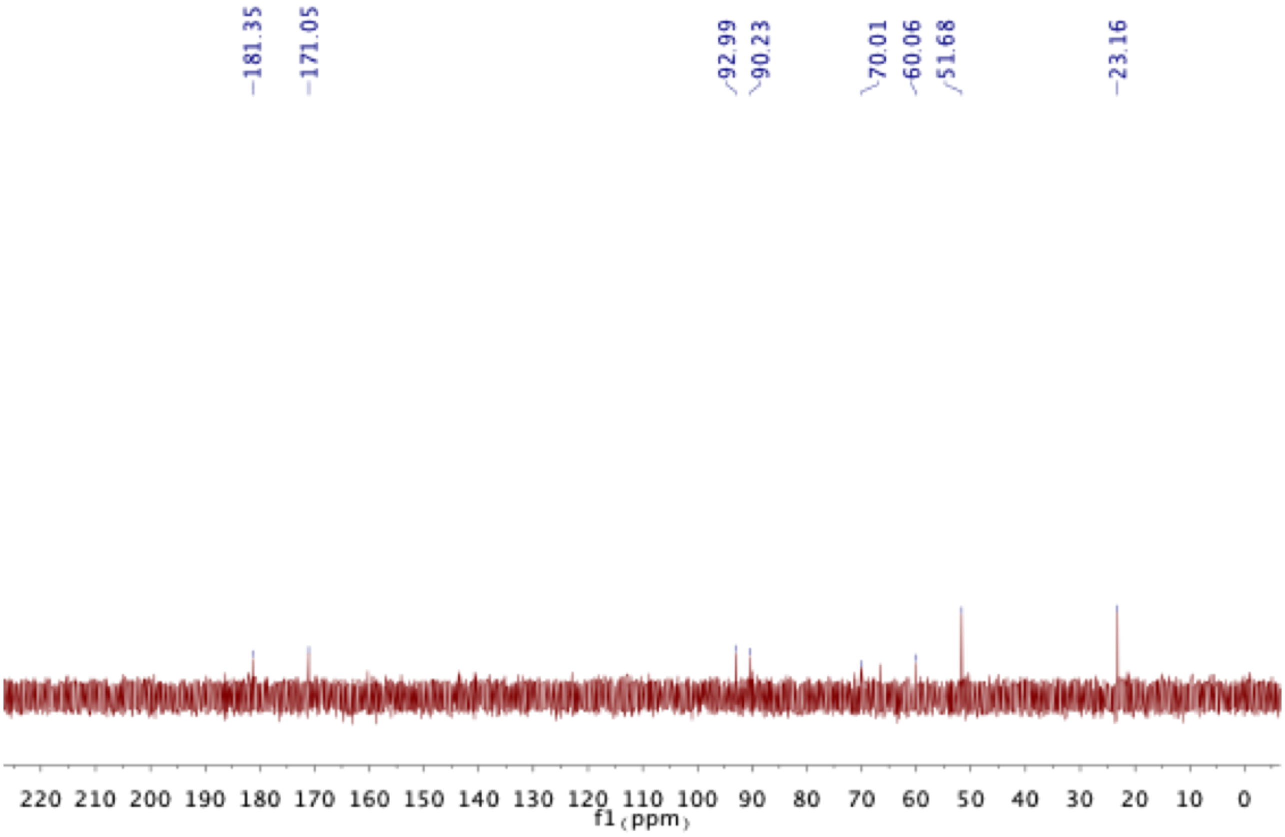

Uridine 5′- Diphospho-(2-azidoacetamido-4-fluoro-1, 2, 4-dideoxy-D-galactopyranosyl) (UDP-4FGalNAz)

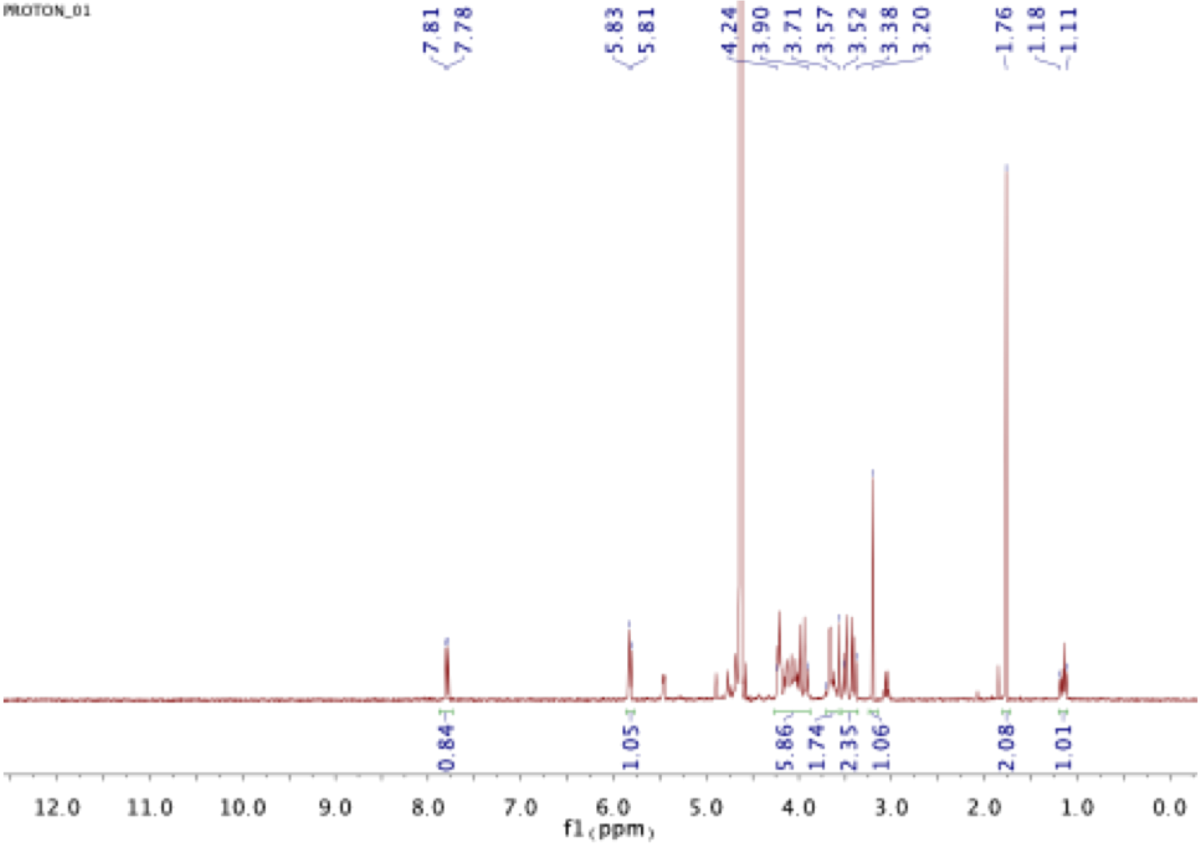
PROTON_01
7.81
7.78
5.83
5.81
4.24
3.90
3.71
3.57
3.52
3.38
3.20
1.76
1.18
1.11
0.84
1.05
5.86
1.74
2.35
1.06
2.08
1.01
12.0 11.0 10.0 9.0 8.0 7.0 6.0 5.0 4.0 3.0 2.0 1.0 0.0
f1 (ppm)

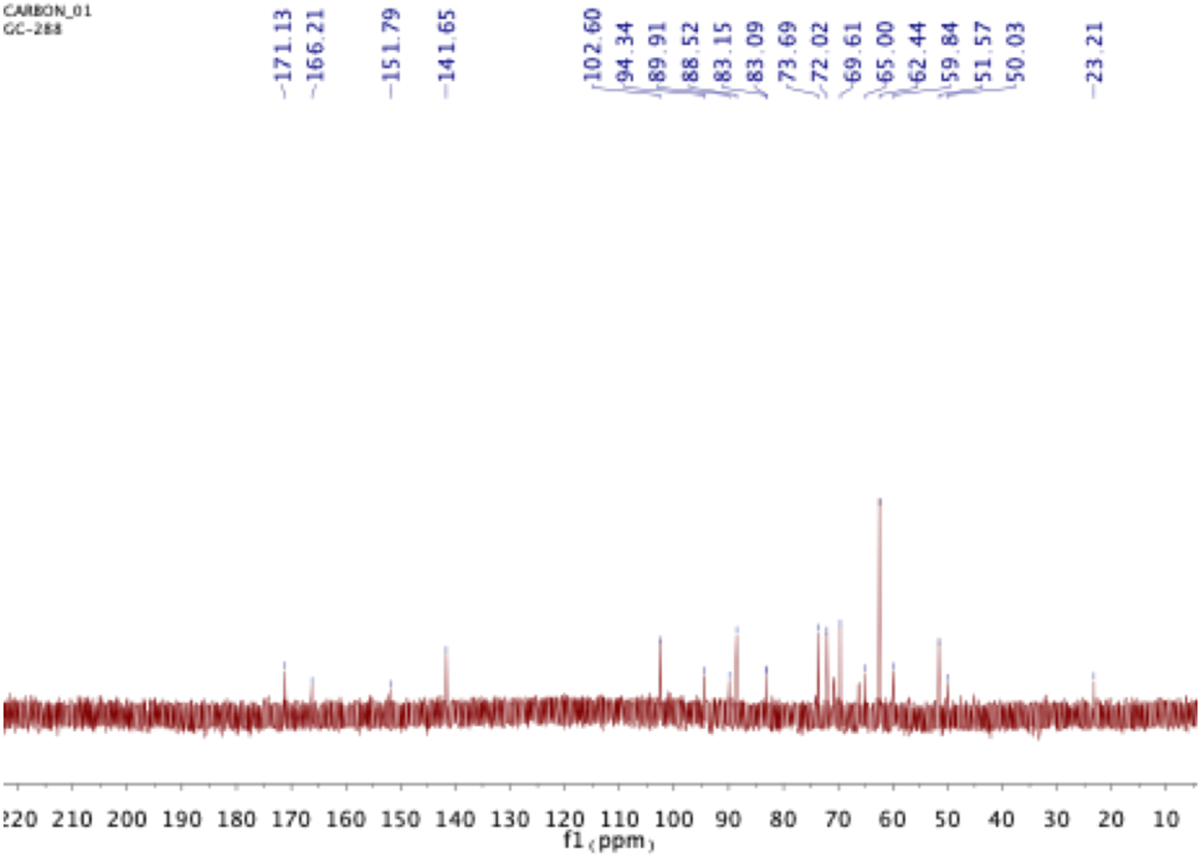
CARBON_01
GC-288
171.13
166.21
151.79
141.65
102.60
94.34
89.91
88.52
83.15
83.09
73.69
72.02
69.61
65.00
62.44
59.84
51.57
50.03
23.21
220 210 200 190 180 170 160 150 140 130 120 110 100 90 80 70 60 50 40 30 20 10
f1 (ppm)

References

(1) Wohnig, S.; Spork, A. P.; Koppermann, S.; Mieskes, G.; Gisch, N.; Jahn, R.; Ducho, C. Total Synthesis of Dansylated Park's Nucleotide for High-Throughput MraY Assays. *Chem - European J* **2016**, *22* (49), 17813–17819.
(2) Bera, S.; Linhardt, R. J. Design and Synthesis of Unnatural Heparosan and Chondroitin Building Blocks. *J Org Chem* **2011**, *76* (9), 3181–3193.
(3) Berkin, A.; Szarek*, W. A.; Kisilevsky*, R. Synthesis of 4-Deoxy-4-Fluoro Analogues of 2-Acetamido-2-Deoxy-d-Glucose and 2-Acetamido-2-Deoxy-d-Galactose and Their Effects on Cellular Glycosaminoglycan Biosynthesis. *Carbohyd Res* **2000**, *326* (4), 250–263.
(4) Sharma, M.; Bernacki, R. J.; Paul, B.; Korytnyk, W. Fluorinated Carbohydrates as Potential Plasma Membrane Modifiers. Synthesis of 4- and 6-Fluoro Derivatives of 2-Acetamido-2-Deoxy-d-Hexopyranoses. *Carbohyd Res* **1990**, *198* (2), 205–221.
(5) Zhai, Y.; Liang, M.; Fang, J.; Wang, X.; Guan, W.; Liu, X.; Wang, P.; Wang, F. NahK/GlmU Fusion Enzyme: Characterization and One-Step Enzymatic Synthesis of UDP-N-Acetylglucosamine. *Biotechnol Lett* **2012**, *34* (7), 1321–1326.
